# Optimized nitrogen rate, plant density, and irrigation level reduced ammonia emission and nitrate leaching on maize farmland in the oasis area of China

**DOI:** 10.7717/peerj.12762

**Published:** 2022-01-19

**Authors:** Aziiba Emmanuel Asibi, Wen Yin, Falong Hu, Zhilong Fan, Zhiwen Gou, Hongwei Yang, Yao Guo, Qiang Chai

**Affiliations:** 1 State Key Laboratory of Aridland Crop Science, Gansu Agricultural University, Lanzhou, China; 2 College of Agronomy, Gansu Agricultural University, Lanzhou, China; 3 Council for Scientific and Industrial Research–Savanna Agricultural Research Institute, Bawku, Ghana

**Keywords:** Environmental sustainability, greenhouse gas, nitrogen application, sustainable cropping

## Abstract

Nitrogen fertilizers play a key role in crop production to meet global food demand. Inappropriate application of nitrogen fertilizer coupled with poor irrigation and other crop management practices threaten agriculture and environmental sustainability. Over application of nitrogen fertilizer increases nitrogen gas emission and nitrate leaching. A field experiment was conducted in China’s oasis irrigation area in 2018 and 2019 to determine which nitrogen rate, plant density, and irrigation level in sole maize (*Zea mays* L.) cropping system reduce ammonia emission and nitrate leaching. Three nitrogen rates of urea (46-0-0 of N-P_2_O_5_-K_2_O), at (N_0_ = 0 kg N ha^−1^, N_1_ = 270 kg N ha^−1^, and N_2_ = 360 kg N ha^−1^) were combined with three plant densities (D_1_ = 75,000 plants/ha^−1^, D_2_ = 97,500 plants/ha^−1^, and D_3_ = 120,000 plants/ha^−1^) with two irrigation levels (W_1_ = 5,250 m_3_/hm^2^ and W_2_ = 4,740 m^3^/hm^2^) using a randomized complete block design. The results showed that, both the main and interaction effects of nitrogen rate, plant density, and irrigation level reduced nitrate leaching (*p* < 0.05). In addition, irrigation level × nitrogen rate significantly (*p* < 0.05) reduced ammonia emission. Nitrate leaching and ammonia emission decreased with higher irrigation level and higher plant density. However, high nitrogen rates increased both nitrate leaching and ammonia emission. The study found lowest leaching (0.35 mg kg^−1^) occurring at the interaction of 270 kg N ha^−1^ × 120,000 plants/ha^−1^ × 4,740 m^3^/hm^2^, and higher plant density of 120,000 plants/ha^−1^ combined with 0 kg N ha^−1^ and irrigation level of 5,250 m^3^/hm^2^ recorded the lowest ammonia emission (0.001 kg N)^−1^. Overall, ammonia emission increased as days after planting increased while nitrate leaching decreased in deeper soil depths. These findings show that, though the contributory roles of days after planting, soil depth, amount of nitrogen fertilizer applied and year of cultivation cannot be undermined, it is possible to reduce nitrate leaching and ammonia emission through optimized nitrogen rate, plant density and regulated irrigation for agricultural and environmental sustainability.

## Introduction

Global food demand is expected to increase from 35 percent to 56 percent between now and 2050, while the global population at risk of famine is expected to increase by eight percent during the same period (van Dijk et al., 2021). Agricultural intensification for food is a problem to environmental sustainability due to the high and overuse of chemical inputs such as synthetic nitrogen (N) (Andraski, Bundy & Brye, 2000). Synthetic N fertilizers have greatly enhanced crop production, however, their long-term use and overuse in agriculture to meet food demand can lead to heavy deposit of nitrate (NO_3_–N) in soils (Zhang et al., 2013; Dai et al., 2016); ammonia (NH_3_–N) emission and soil salinity (David et al., 2009). The Intergovernmental Panel on Climate Change (IPCC) states that an average of 14% N emission occurs from 11.2–15.7 million tons of N fertilizer applied (Bouwman, Boumans & Batjes, 2002; De Klein, Smith & Monaghan, 2006). A significant amount of N is lost to the atmosphere in the form of ammonia from applied N fertilizers and it is a key contributor to NH_3_–N gas emission (Zhang et al., 2011; Ma et al., 2021). Ammonia gas originating from agricultural production systems have negative environmental impacts (Gruber & Galloway, 2008) and plays a key role in the local atmospheric conditions (Zhang et al., 2008). In the oxidation of ammonium sulfate to nitric and sulfuric acid, ammonia gas plays a crucial role and is a major constituent of the formation of atmospheric particulate matter and secondary aerosols (Wang et al., 2020; Feng et al., 2021), which has a negative effect on human and ecosystem health (Powlson et al., 2008). Ammonia emission from N application is a major loss route of applied N (Harrison & Webb, 2001; Pacholski et al., 2008).

In China, ammonia emission from N application represents 46.1% of total emission (Zhang et al., 2011), and the current rise in N fertilizer application is a major cause of the ammonia emission (Ju et al., 2004; Zheng et al., 2002). In intensive agricultural production systems, up to 75% of applied N is not used by the plant but lost through leaching (Hodge, Robinson & Fitter, 2000; Asghari & Cavagnaro, 2011). Nitrate leaching can lead to groundwater contamination due to inappropriate N application (Kaushal et al., 2011; Perego et al., 2012). Leached N can damage both surface and groundwater resources and make it unsafe for human and animal consumption (Barton & Colmer, 2006). Groundwater pollution by nitrate have been associated with excessive N application from agricultural systems (Jalali, 2005; Wakida & Lerner, 2005). The application of N at rates above optimum can cause nitrate leaching into groundwater and this can limit N availability to crops (Li et al., 2015a). Though nitrate accumulation and leaching in agricultural soils differ, it is largely influenced by N application time and rate, precipitation, soil types, and the cropping system (Yang et al., 2004; Fang et al., 2006). Urea hydrolysis is considerably limited in a low-moisture environment and can lead to a great loss of N through ammonia emissions (Klimczyk, Siczek & Schimmelpfennig, 2021). Precipitation has a close connection with NH_3_–N emissions (Li et al., 2015b; Abdo et al., 2021). Rainfall of up to 3 mm nearly after fertilizer application can increase NH_3_–N emission, while precipitation of 71.4 mm can reduce ammonia emission by 84.0% (Sanz-Cobena et al., 2011). High soil moisture is not favorable for ammonia emission; low moisture levels increase NH_3_–N emission (Han, Zhou & Wang, 2014; Han et al., 2016). Plant density and the management of different crops on farms can directly or indirectly influence ammonia emission (Zhan et al., 2021; Adalibieke et al., 2021).

In China, N application rate have reached 450 kg N ha^−1^ per year, escalating the risks of groundwater pollution (Li et al., 2005). Nitrogen rates that exceed the environmentally optimal nitrogen rate, must be reduced to avoid environmental damages, and this can be done while still meeting China’s food need (Zhang et al., 2018). Nitrate leaching and accumulation is found to reach 1.88–15.7 kg N ha^−1^ at 60 cm soil layer with N fertilizer rate increasing from 0 to 360 kg N ha^−1^ in China (Liang et al., 2011). Nitrogen rates from 0–320 kg N ha^−1^ with higher precipitation is found to leach NO_3_–N from 40 to 300 cm soil depth (Dai et al., 2016), this suggests that with continuous N application and cropping N is leached to deeper soil layers. Appropriate plant density, time of sowing, and crop selection can reduce NO_3_–N leaching (Hashimoto et al., 2007). Leaching of NO_3_–N into soil layers beyond crop roots is a key N loss route in the cropping system (Cui et al., 2014; Li et al., 2016). Nitrate leaching, ammonia and nitrous oxide emission, and run-off can reduce N accessibility to plants (Liang et al., 2017). Higher irrigation and N rates may escalate NO_3_–N leaching into freshwater resources (Jia et al., 2014; Gentry, David & McIsaac, 2014). In many humid areas, heavy precipitation can cause NO_3_–N leaching while excessive irrigation can facilitate leaching in arid and semi-arid areas (Jalali, 2005). Even though, variable and insufficient irrigation scheduling can reduce NO_3_–N leaching, irrigation scheduling is a challenging task to farmers (Barton & Colmer, 2006). Soil NO_3_–N accumulation from subsurface drainage resulting from irrigation is an important factor that affects NO_3_–N leaching (Tamini & Mermoud, 2002). When the quantity of water supplied through irrigation does not meet the evapotranspiration needs of crops, the application of N to fully irrigated environments could prompt N over-application, thereby increasing N losses to groundwater (Tarkalson et al., 2006).

Maize cultivation in China requires greater N application to obtain higher grain and biomass yields (Ren et al., 2021). When N application exceeds crop requirements, N accumulation and leaching are likely undesirable outcomes (Djaman et al., 2013; Yang, Lu & Yin, 2015). Many studies have mostly focused on the optimal N rate to improve N use efficiency and increase yield to its highest (Xu et al., 2014), by testing the soil NO_3_−N content in the root zone (Cui et al., 2010), and recommending fertilizer application based on soil test, grain yield and crop responses (He et al., 2009). Very few studies have attempted to evaluate N rate, plant density and irrigation management with regards to ammonia emission and nitrate leaching.

Ammonia emission and nitrate leaching are often neglected in most intensive cropping systems and are not strictly regulated in many countries. Nitrogen losses are key threats to environmental sustainability. The application of N fertilizers facilitates N_2_O effluxes and its production significantly increases when N application is higher; for example, 380 kg N ha^−1^ can produce more emission than 250 kg N ha^−1^ particularly after fertilization (Nan et al., 2016). Therefore, the quantification of current N fertilization and improved N management practices and policies in Chinese agriculture is of national and world interest (Wu et al., 2015). The objectives of this study were to identify which nitrogen rate, plant density, and irrigation level reduces ammonia emission and nitrate leaching to improve N management for sustainable cereal production, food security, and environmental sustainability.

## Materials and Methods

### Experimental location

**Figure 1:**
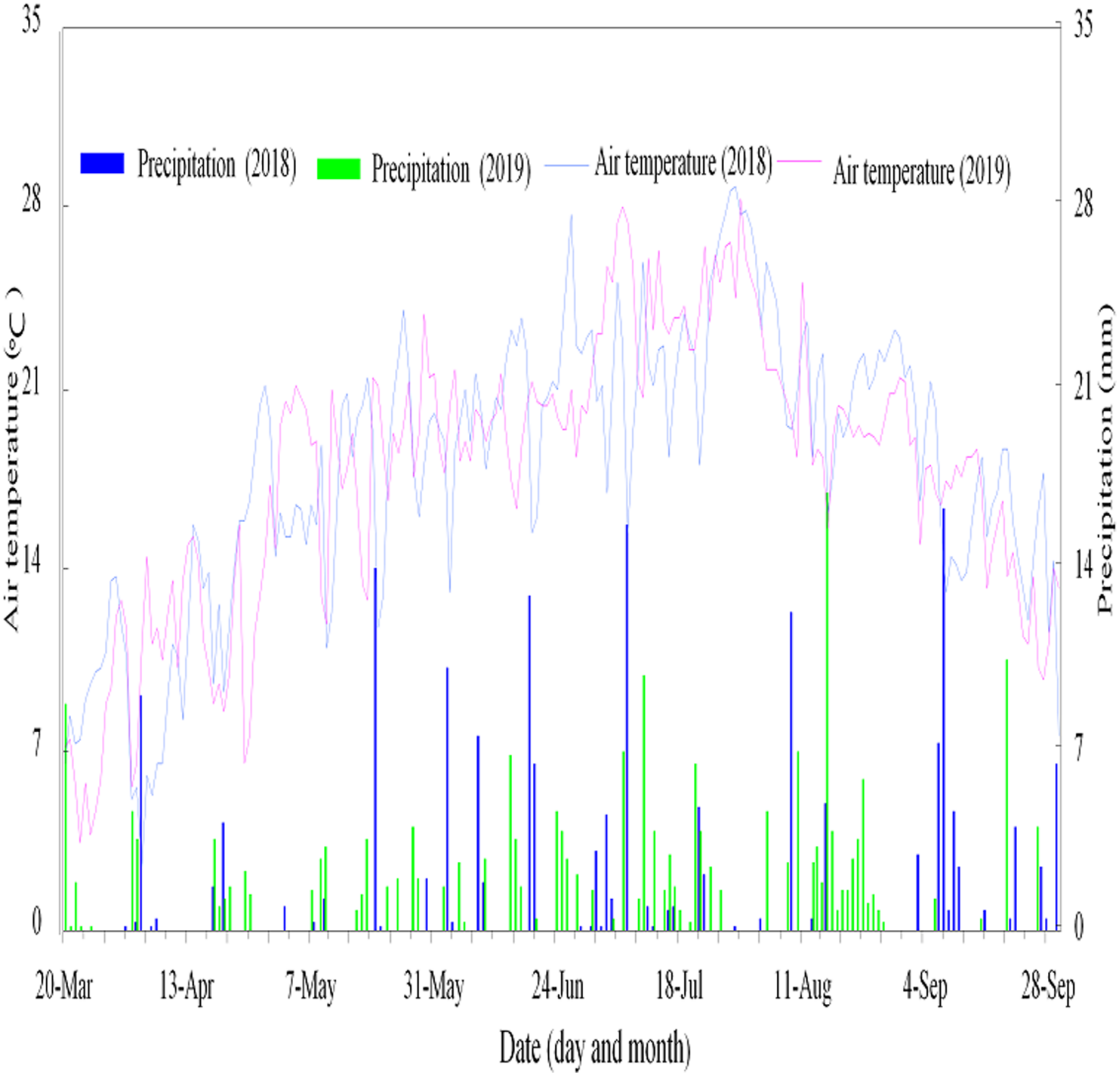
Air temperature and precipitation at the experimental site during the study period (2018 and 2019). Air temperature and precipitation at the experimental site during the study period (2018 and 2019).

The experiment was conducted at Wuwei, Gansu Agricultural University Experiment field in 2018 and 2019, from April to September, on 37°96′N, 102°64′E altitude, and 1,776 m above sea level. The experiment station is situated in the eastern part of the Hexi corridor of Northwestern China with a temperate and arid climate. Soils in this area are classified as Aridisol; a type of calcareous desert soil (Shao et al., 2021). Average annual sunshine duration, air temperature, precipitation, and potential evaporation in the location are 2,945 h, 7.2 °C, 150 mm, and 2,400 mm, respectively. Daily precipitation and air temperature were obtained for the experiment in both years by the climate data sub-station of Wuwei, 100 m away from the experiment field (Fig. 1). Soil ammonium nitrogen (NH_4_^+^–N), soil nitrate-nitrogen (NO_3_–N), soil total nitrogen (TN), and soil bulk density by soil depth-layers before the start of the experiment in April of 2018 and 2019 are shown in (Table 1). The experimental field was monocropped with maize the previous year before this experiment was conducted.

**Table 1:** Ammonium nitrogen (NH_4_–N), nitrate-nitrogen (NO_3_–N), and total nitrogen (TN), bulk density 2018 and 2019. Soil ammonium nitrogen (NH_4_–N), nitrate-nitrogen (NO_3_–N), and total nitrogen (TN) by soil depth layer before the start of the experiment in 2018 and 2019.

	2018				2019			
Soil depth (cm)	NH_4_–N	NO_3_–N	TN	Bulk density	NH_4_–N	NO_3_–N	TN	Bulk density
	mg kg^−1^	mg kg^−1^	g kg^−1^	g cm^−3^	mg kg^−1^	mg kg^−1^	g kg^−1^	g cm^−3^
0–30	11.085	3.665	1.550	1.259	15.913	4.242	1.600	1.251
30–60	11.023	3.747	1.500	1.240	15.707	4.993	1.710	1.243
60–90	11.000	3.575	1.000	1.180	16.077	5.567	1.500	1.184
90–120	11.945	3.558	1.400	1.210	15.560	4.820	1.420	1.200
120–150	11.354	3.232	1.240	1.160	11.875	3.773	1.210	1.170

### Experimental layout

The experiment was a 3 × 3 × 2 factorial experiment in a randomized complete block design (RCBD) with three replicates. Each treatment had a plot measuring 7 × 5 m and was separated by 80 cm. A 50 cm ridge was built between plots to reduce the potential of water movement between plots. The treatment comprises three N rates of urea (46–0–0 of N–P_2_O_5_–K_2_O) (N_0_ = 0 kg N ha^−1^, N_1_ = 270 kg N ha^−1^, and N_2_ = 360 kg N ha^−1^), three maize plant densities (D_1_ = 75,000 plants/ha^−1^, D_2_ = 97,500 plants/ha^−1^, and D_3_ = 120,000 plants/ha^−1^), and spaced at 40 × 33 cm, 40 × 26 cm, and 40 × 21 cm to get the required plant densities respectively, and combined with two irrigation levels (W_1_ = 5,250 m^3^/hm^2^ and W_2_ = 4,740 m^3^/hm^2^). Drip irrigation lines with valves were connected to water meters to regulate and measure the amount of water irrigated.

### Field management practices

A moldboard plough was used to till soil to a depth of 20 cm in April of both years, followed by a rotary tiller to loosen soil lumps. Nitrogen application was applied in triplicates, with 20% of the total N broadcasted and incorporated to 20 cm soil depth using rotary tiller prior to maize seeding, and the remaining split into two portions. It was applied by deep placement into the soil at the nine-leaf collar stage and 15 days after flowering. A white plastic film of (120 cm wide and 0.01 mm thickness) was mulched on the plots before maize seeding was done each year. Maize cultivar (Xianyu 335) was sown on 17th and 19th April 2018 and 2019 using a hand-held pressure-inject planter. Irrigation was done at 90, 120, and 90 mm at the nine, fourteen leaf, and grain-filling stages, respectively, using drip irrigation lines. Water meters were connected to the drip lines to measure the amount of water irrigated. Weeds were controlled by hand throughout the crop growing period in both years. No chemical application for weed and pest control and no diseases and pests were recorded in the experiment.

### Ammonia gas sampling and analysis

The measurement of ammonia emission was done as described in Wang et al. (2005), Akbari et al. (2020). The polyvinyl chloride rigid measuring container is a plastic tube with an interior diameter and height measuring 15 and 12 cm respectively. Two-disc foams 2 cm of 2 cm thickness and 16 cm diameter were uniformly submerged in 20 ml solution of glycerol phosphate (40 ml of glycerol + 50 ml of phosphoric acid, and the volume was adjusted to 1,000 ml). The two layers of disc foams were positioned in the firm polyvinyl chloride rigid tube so that the lower layer of the disc foam is 5 cm from the ground and the upper layer of the disc foam is at the level with the top of the tube. Ammonia emission was believed to start on the day of fertilization, sowing, and during the crop growth period, and after crop harvest. Therefore measurements started immediately after sowing. The gas was extracted by putting the disc foam into a 150 ml sealed bag and adding 100 ml of Sodium chloride and shook for an hour. The samples were filtered, and 5 ml of the filtrate was transferred to a 50 ml volumetric flask, 5 ml of Sodium chloride was added to the 50 ml volumetric flask containing the sample filtrate. Phenol liquor, Sodium hydroxide aqueous of 5 ml and 1 ml of masking agent was added and allowed to stand for an hour before distilled water was filled to the volumetric flask mark. The samples were analyzed using a mass spectrophotometer. The machine was switched on for 20 min in advance to warm and adjusted to 625.0 nm. Daily (mg day^−1^) and cumulative NH_3_–N losses (kg N ha^−1^) were calculated to express the N loss in relation to the N applied.

### Soil sampling for nitrate and analysis

Soil samples were collected from 0–30, 30–60, 60–90, 90–120, and 120–150 cm soil layers using a soil sampling auger (internal diameter of 4.0 cm) from each plot immediately preceding maize sowing, during and after maize harvest in each year. The interval and days for the sampling were done before planting and at 30, 60, 90, 120, and 150 days after planting. The samples were air-dried using the oven drying method (O’Kelly, 2004) and ground into fine powder and sieved through (<2 mm) wire mesh for the chemical analysis. Soil TN was determined by the standard Semi-micro–Kjeldahl method (Bao, 1999). Soil NH_4_^+^–N and NO_3_–N were determined by the spectrophotometry method with a Discrete Auto Analyzer (SMARTChem 450, Beijing, China), (NATESC, 2006). The leached and accumulated N (LAN) was calculated using the following equation (Dai et al., 2016):

${\rm LAN = Ti \times Di \times Ci \text / 10}$

where Ti and Di represents the thickness and bulk density of the soil layer at (30 cm), (g cm^−3^), respectively, Ci represents soil NO_3_–N leached or accumulated (mg kg^−1^) of the corresponding layer, and 10 represents the conversion coefficient.

### Statistical Analyses

Analysis of variance and effect was conducted using the general linear model function in Genstat statistical software (version 12., Chicago, IL, USA) to determine whether N application rate, plant density, and irrigation levels influence, NH_3_–N emission and NO_3_–N leaching and their interactive effects. Differences among means were tested with Fisher’s protected least significant difference at *p* ≤ 0.05.

## Results

### Soil ammonium nitrogen, nitrate-nitrogen, total nitrogen, bulk density, precipitation, and temperature before the start of the experiment in 2018 and 2019

The soil at the experiment site is coarse sandy clay and classified as aridisol, a desert soil with calcareous particles (Table 1) (Shao et al., 2021). The lowest NH_4_^+^–N was recorded in 2018 (11.945 mg kg^−1^) while the highest NH_4_^+^–N was recorded in 2019 (16.077 mg kg^−1^) at 60–90 soil depth. The lowest NO_3_–N was recorded at 120–150 cm soil depth (3.232 mg kg^−1^) in 2018 while the highest NO_3_–N (5.567 mg kg^−1^) was recorded at 60–90 cm soil depth in 2019. At 120 and 150 cm soil depths in both years, there was no much variation in NH_4_^+^–N and NO_3_–N concentration but differed to other layers. This variation in NH_4_^+^–N and NO_3_–N in the experimental soil may be partly attributed to the varying precipitation and air temperature (Fig. 1).

### Influence of nitrogen fertilization, plant density, and irrigation regime on nitrate accumulation and leaching

**Figure 2:**
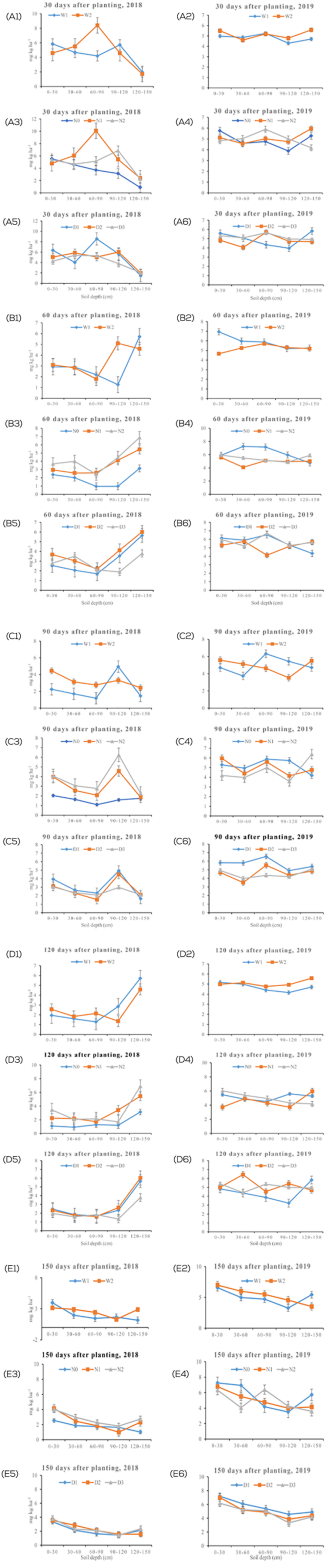
Effect of individual treatment levels on NO_3_–N leaching and accumulation in maize farmland across the experiment years. Effect of individual treatment levels on NO_3_–N leaching and accumulation in maize farmland across the experiment years. (A, B) Irrigation (water level) in 2018 and 2019, respectively, where W_1_ = 5,250 m^3^/hm^2^ and W_2_ = 4,740 m^3^/hm^2^. (C, D) Nitrogen fertilizer levels in 2018 and 2019, respectively, where N_0_ = 0 kg N ha^−1^, N_1_ = 270 kg N ha^−1^, and N_2_ = 360 kg N ha^−1^, while (E, F) Represent maize plant density levels in 2018 and 2019, respectively, D_1_ = 75,000 plants/ha^−1^, D_2_ = 97,500 plants/ha^−1^, and D_3_ = 120,000 plants/ha^−1^. The error bars represent standard error of means from the triplicate readings.

The effect of nitrogen fertilizer rate, plant density and irrigation level on NO_3_–N leaching and accumulation at the various sampling depths and days after planting over the 2 years are shown in Figs. 2A, 2B, 2C, 2D & 2E and Tables 2, 3, 4, and 5. It was found that the factors had marked individual and interactive effects on leaching and the accumulation of nitrate in the soil. Irrigation level significantly (*p* < 0.05) affected the leaching and accumulation of nitrate in the soil. Nitrate accumulation relatively increased with a decrease in irrigation level for the first three soils depths but increased correspondingly at 90–120 cm and 120–150 cm especially at 30 days through to 120 days after planting in 2018. In 2019 however, the amount of nitrate accumulated in the soil was generally higher at 4,740 m^3^/hm^2^ (W_2_) except for 60 days after planting where the higher (W_1_) irrigation level resulted in more leaching of nitrate (Figs. 2A, 2B, 2C, 2D & 2E). With regard to N rate main effect, considerable (*p* < 0.05) variation occurred in relation to leaching and accumulation of the nitrate. Accumulation increased as higher amount of N fertilizer was applied with exception of 90 days after planting in 2019 where the reverse occurred at the first four soil depths (Figs. 2A, 2B, 2C, 2D, & 2E). A significant effect was also recorded in relation to plant density, with a general increase in nitrate accumulation at the lower plant densities as compared to the highest density (D_3_) (Figs. 2A, 2B, 2C, 2D, & 2E) in both years. In terms of interaction effects, all two-factor and three-factor combinations generally were significant (*p* < 0.05) and affected leaching and accumulation regardless of the soil depth, duration after planting and year of cultivation. However, no linear or specific trends were observed to that effect. Nonetheless, the lowest (0.35 mg kg^−1^) leaching and accumulation occurred at the combination of 270 kg N ha^−1^, 120,000 plants/ha^−1^ and 4,740 m^3^/hm^2^ at 90–120 cm soil depth at 150 days after planting in 2018 while the highest (9.94 mg kg^−1^) occurred at the combination of 0 kg N ha^−1^, 75,000 plants/ha^−1^ and 4,740 m^3^/hm^2^ at 90–120 cm soil depth at 60 days after planting in 2019.

**Table 2:** Combined effect of irrigation (water level) and N fertilizer rates on NH_3_ emission across the 2 years in maize farmland. Irrigation (water level) comprised W_1_ = 5,250 m^3^/hm^2^ and W_2_ = 4,740 m^3^/hm^2^ and nitrogen fertilizer levels consisted of N_0_ = 0 kg N ha^−1^, N_1_ = 270 kg N ha^−1^, and N_2_ = 360 kg N ha^−1^. Means separation was done with LSD at *p*-value < 0.05. Means ± SEMs with a common superscript indicates no significant difference at *p* < 0.05 and those with otherwise are significantly different at *p* < 0.05.

		30 days after planting (2018)					30 days after planting (2019)				
Water level	N rate	0–30 cm	30–60 cm	60–90 cm	90–120 cm	120–150 cm	0–30 cm	30–60 cm	60–90 cm	90–120 cm	120–150 cm
	N_0_	6.98 ± 0.42^a^	3.92 ± 0.57^b^	4.17 ± 0.53^b^	3.47 ± 0.76^b^	0.94 ± 0.15^c^	4.59 ± 0.48^a^	4.50 ± 1.02	5.95 ± 0.43^b^	4.33 ± 0.17^bc^	5.66 ± 0.54^a^
W_1_	N_1_	4.66 ± 0.61^bc^	4.84 ± 0.66^ab^	4.27 ± 0.65^b^	7.39 ± 0.95^a^	3.31 ± 0.82^a^	5.21 ± 0.51^ab^	5.04 ± 0.52	4.89 ± 0.09^c^	4.64 ± 0.28^b^	5.57 ± 0.66^a^
	N_2_	5.93 ± 0.91^ab^	5.30 ± 0.93^ab^	4.17 ± 0.60^b^	6.32 ± 0.80^a^	1.70 ± 0.33^bc^	5.15 ± 0.99^ab^	5.05 ± 0.37	4.93 ± 0.27^c^	3.94 ± 0.77^bc^	2.88 ± 0.45^b^
	N_0_	4.09 ± 0.57^c^	5.22 ± 0.82^ab^	3.23 ± 0.57^b^	2.81 ± 0.41^b^	0.82 ± 0.13^c^	6.94 ± 0.74^b^	4.69 ± 1.01	3.57 ± 0.79^d^	3.45 ± 0.43^c^	4.92 ± 0.87^a^
W_2_	N_1_	4.92 ± 0.38^bc^	7.27 ± 0.54^a^	15.88 ± 5.93^a^	3.52 ± 0.49^b^	1.49 ± 0.23^bc^	5.03 ± 0.97^ab^	4.10 ± 0.42	5.16 ± 0.35^bc^	4.86 ± 0.21^b^	6.35 ± 0.57^a^
	N_2_	4.79 ± 0.46^bc^	4.03 ± 0.72^b^	6.11 ± 0.80^b^	7.44 ± 0.84^a^	2.86 ± 0.53^ab^	4.55 ± 0.43^a^	5.02 ± 0.95	6.87 ± 0.41^a^	6.08 ± 0.39^a^	5.49 ± 0.34^a^
*p*-value		0.002	0.008	0.013	<0.001	<0.001	0.041	0.286	<0.001	<0.001	<0.001
Lsd		1.20	1.64	6.03	1.38	0.93	1.74	1.06	0.59	0.77	0.97
		**60 days after planting (2018)**					**60 days after planting (2019)**				
**Water level**	**N rate**	**0–30 cm**	**30–60 cm**	**60–90 cm**	**90–120 cm**	**120–150 cm**	**0–30 cm**	**30–60 cm**	**60–90 cm**	**90–120 cm**	**120–150 cm**
	N_0_	2.73 ± 0.38	2.63 ± 0.51^bc^	0.98 ± 0.15^c^	0.83 ± 0.20^b^	3.47 ± 0.76^b^	7.51 ± 0.44	6.71 ± 0.38^ab^	7.46 ± 0.62	4.96 ± 0.39^ab^	5.95 ± 0.43^b^
W_1_	N_1_	2.97 ± 0.80	2.69 ± 0.51^bc^	3.69 ± 0.92^a^	1.77 ± 0.53^b^	7.39 ± 0.95^a^	6.00 ± 0.36	5.38 ± 0.41^b^	5.29 ± 0.56	6.04 ± 0.61^ab^	4.89 ± 0.09^c^
	N_2_	3.05 ± 0.48	3.38 ± 0.81^ab^	1.90 ± 0.44^bc^	1.22 ± 0.40^b^	6.32 ± 0.80^a^	7.35 ± 0.44	5.84 ± 0.57^ab^	4.91 ± 0.74	4.61 ± 0.62^b^	4.93 ± 0.27^c^
	N_0_	2.03 ± 0.56	1.40 ± 0.18^c^	0.94 ± 0.22^c^	1.12 ± 0.24^b^	2.81 ± 0.31^b^	4.33 ± 0.83	7.81 ± 0.41^a^	6.85 ± 0.79	6.94 ± 1.00^a^	3.57 ± 0.79^d^
W_2_	N_1_	2.93 ± 0.62	2.47 ± 0.59^bc^	1.50 ± 0.24^bc^	6.47 ± 1.26^a^	3.52 ± 0.49^b^	5.15 ± 0.94	2.81 ± 0.69^c^	4.91 ± 0.80	3.87 ± 0.55^b^	5.16 ± 0.35^bc^
	N_2_	4.33 ± 0.47	4.63 ± 0.87^a^	2.93 ± 0.56^ab^	7.73 ± 0.76^a^	7.44 ± 0.84^a^	4.53 ± 0.97	5.17 ± 0.64^b^	5.37 ± 0.98	5.13 ± 0.82^ab^	6.87 ± 0.41^a^
*p*-value		0.151	0.026	<0.001	<0.001	<0.001	0.218	0.002	0.464	0.002	<0.001
Lsd		1.45	1.26	0.97	1.02	1.38	2.02	1.38	1.28	1.54	0.59
		**90 days after planting (2018)**					**90 days after planting (2019)**				
**Water level**	**N rate**	**0–30 cm**	**30–60 cm**	**60–90 cm**	**90–120 cm**	**120–150 cm**	**0–30 cm**	**30–60 cm**	**60–90 cm**	**90–120 cm**	**120–150 cm**
	N_0_	1.68 ± 0.56^c^	0.83 ± 0.14^d^	0.92 ± 0.24^c^	1.05 ± 0.16^b^	1.30 ± 0.19^c^	5.66 ± 0.54^a^	4.19 ± 0.62^ab^	6.51 ± 0.87^a^	6.77 ± 0.69	3.21 ± 0.15^d^
W_1_	N_1_	3.27 ± 0.56^bc^	1.31 ± 0.22^cd^	1.46 ± 0.29^bc^	6.82 ± 1.12^a^	1.85 ± 0.44^bc^	5.57 ± 0.66^a^	3.67 ± 0.54^ab^	5.55 ± 0.74^a^	4.48 ± 0.76	3.44 ± 0.53^cd^
	N_2_	1.80 ± 0.44^c^	2.99 ± 0.39^ab^	1.18 ± 0.19^c^	6.98 ± 1.08^a^	1.23 ± 0.19^c^	2.88 ± 0.45^b^	3.37 ± 0.63^b^	6.93 ± 0.70^a^	5.08 ± 0.54	7.52 ± 0.44^a^
	N_0_	2.38 ± 0.24^c^	2.45 ± 0.28^bc^	1.27 ± 0.25^bc^	2.13 ± 0.67^b^	2.20 ± 0.07^b^	4.92 ± 0.87^a^	5.70 ± 1.00^a^	5.25 ± 0.79^ab^	4.68 ± 0.87	5.16 ± 0.50^bcd^
W_2_	N_1_	4.68 ± 0.51^ab^	3.82 ± 0.37^a^	2.68 ± 0.53^b^	2.33 ± 0.72^b^	1.85 ± 0.29^bc^	6.35 ± 0.57^a^	5.09 ± 0.58^ab^	5.60 ± 0.56^a^	3.82 ± 0.45	6.07 ± 0.35^ab^
	N_2_	6.32 ± 0.71^a^	3.13 ± 0.40^ab^	4.35 ± 0.69^a^	5.52 ± 0.42^a^	3.27 ± 0.17^a^	5.49 ± 0.34^a^	4.58 ± 0.21^ab^	3.06 ± 0.21^b^	2.10 ± 0.23	5.27 ± 0.74^bc^
*p*-value		0.001	0.002	0.001	<0.001	<0.001	<0.001	0.954	0.003	0.093	<0.001
Lsd		1.46	0.87	0.97	1.00	0.53	0.97	1.45	1.55	1.50	1.35
		**120 days after planting (2018)**					**120 days after planting (2019)**				
**Water level**	**N rate**	**0–30 cm**	**30–60 cm**	**60–90 cm**	**90–120 cm**	**120–150 cm**	**0–30 cm**	**30–60 cm**	**60–90 cm**	**90–120 cm**	**120–150 cm**
	N_0_	0.95 ± 0.21	0.77 ± 0.17	0.98 ± 0.15	1.43 ± 0.05^b^	3.47 ± 0.76^b^	4.57 ± 0.44^a^	5.16 ± 0.28^bc^	4.36 ± 0.68	4.95 ± 0.61^ab^	5.66 ± 0.54^a^
W_1_	N_1_	1.86 ± 0.43	1.99 ± 0.46	1.27 ± 0.27	5.59 ± 1.24^a^	7.39 ± 0.95^a^	5.23 ± 0.78^a^	5.95 ± 1.00^ab^	3.92 ± 0.41	2.74 ± 0.65^b^	5.57 ± 0.66^a^
	N_2_	3.03 ± 0.47	2.05 ± 0.19	1.60 ± 0.45	1.52 ± 0.39^b^	6.32 ± 0.80^a^	5.69 ± 0.69^a^	3.88 ± 0.45^c^	4.95 ± 0.37	4.80 ± 1.10^ab^	2.88 ± 0.45^b^
	N_0_	1.24 ± 0.15	1.06 ± 0.19	1.57 ± 0.27	0.97 ± 0.21^b^	2.81 ± 0.31^b^	6.37 ± 0.24^a^	4.53 ± 0.45^bc^	4.69 ± 0.56	6.25 ± 0.60^a^	4.92 ± 0.87a
W_2_	N_1_	2.60 ± 0.36	2.37 ± 0.38	2.18 ± 0.41	1.24 ± 0.21^b^	3.52 ± 0.49^b^	2.22 ± 0.23^b^	3.95 ± 0.70^c^	4.67 ± 0.90	4.70 ± 0.96^ab^	6.35 ± 0.57^a^
	N_2_	3.84 ± 0.38	2.08 ± 0.36	2.66 ± 0.41	1.88 ± 0.29^b^	7.44 ± 0.84^a^	6.38 ± 0.70^a^	6.94 ± 0.74^a^	4.93 ± 1.02	3.85 ± 0.74^b^	5.49 ± 0.34^a^
*p*-value		0.715	0.785	0.609	<0.001	<0.001	<0.001	<0.001	0.753	0.023	<0.001
Lsd		0.99	0.73	0.69	1.09	1.38	1.44	1.11	1.46	1.51	0.97
		**150 days after planting (2018)**					**150 days after planting (2019)**				
**Water level**	**N rate**	**0–30 cm**	**30–60 cm**	**60–90 cm**	**90–120 cm**	**120–150 cm**	**0–30 cm**	**30–60 cm**	**60–90 cm**	**90–120 cm**	**120–150 cm**
	N_0_	2.83 ± 0.48^bc^	1.40 ± 0.28	1.30 ± 0.19^c^	1.75 ± 0.08^a^	0.87 ± 0.22^b^	7.44 ± 0.44	5.76 ± 0.28	3.21 ± 0.68^d^	3.21 ± 0.61^bc^	6.77 ± 0.54
W_1_	N_1_	4.17 ± 0.39^ab^	2.26 ± 0.41	1.85 ± 0.44^bc^	1.44 ± 0.13^a^	1.41 ± 0.29^b^	5.86 ± 0.78	5.37 ± 1.00	3.44 ± 0.41^cd^	2.39 ± 0.65^c^	4.48 ± 0.66
	N_2_	4.95 ± 0.33^a^	2.23 ± 0.35	1.23 ± 0.19^c^	1.78 ± 0.10^a^	1.24 ± 0.20^b^	6.46 ± 0.69	3.86 ± 0.45	7.52 ± 0.37^a^	4.23 ± 1.10^b^	5.08 ± 0.45
	N_0_	2.27 ± 0.27^c^	2.33 ± 0.40	2.20 ± 0.06^b^	1.53 ± 0.20^a^	1.17 ± 0.25^b^	7.08 ± 0.24	8.12 ± 0.45	5.16 ± 0.56^bcd^	3.79 ± 0.60^b^	4.68 ± 0.87
W_2_	N_1_	4.10 ± 0.53^ab^	2.67 ± 0.41	1.85 ± 0.29^bc^	0.55 ± 0.13^b^	3.23 ± 0.55^a^	7.71 ± 0.23	5.64 ± 0.70	6.07 ± 0.90^ab^	5.61 ± 0.96^a^	3.82 ± 0.57
	N_2_	3.10 ± 0.36^bc^	3.72 ± 0.28	3.27 ± 0.17^a^	1.84 ± 0.10^a^	4.25 ± 0.68^a^	6.22 ± 0.69	4.27 ± 0.74	5.27 ± 1.02^bc^	4.28 ± 0.74^ab^	2.10 ± 0.34
*p*-value		0.025	0.289	<0.001	0.002	0.006	0.104	0.052	<0.001	<0.001	0.093
Lsd		0.92	0.96	0.53	0.36	1.13	1.62	1.31	1.35	0.93	1.50

**Note:**

Irrigation (water level) comprised W_1_ = 5,250 m^3^/hm^2^ and W_2_ = 4,740 m^3^/hm^2^ and nitrogen fertilizer levels consisted of N_0_ = 0 kg N ha^−1^, N_1_ = 270 kg N ha^−1^, and N_2_ = 360 kg N ha^−1^. Values (mean ± standard error of mean) with different superscripts in the same column are significantly different at *p* < 0.05.

**Table 3:** Combined effect of irrigation (water level) and plant density on NH_3_–N emission across the 2 years in maize farmland. Irrigation (water level) comprised W_1_ = 5,250 m^3^/hm^2^ and W_2_ = 4,740 m^3^/hm^2^ and maize plant density included D_1_ = 75,000 plants/ha^−1^, D_2_ = 97,500 plants/ha^−1^, and D_3_ = 120,000 plants/ha^−1^. Means separation was done with least significant difference at *p*-value < 0.05. Means ± SEMs with a common superscript indicates no significant difference at *p* < 0.05 and those with otherwise are significantly different at *p* < 0.05.

		30 days after planting (2018)					30 days after planting (2019)				
Water level	Plant density	0–30 cm	30–60 cm	60–90 cm	90–120 cm	120–150 cm	0–30 cm	30–60 cm	60–90 cm	90–120 cm	120–150 cm
	D_1_	6.48 ± 0.85^a^	2.65 ± 0.34^b^	2.97 ± 0.28^b^	5.74 ± 1.20^ab^	1.44 ± 0.38^ab^	5.46 ± 0.46^a^	6.62 ± 0.62^a^	5.32 ± 0.24^bc^	3.74 ± 0.35^c^	4.58 ± 0.87^bc^
W_1_	D_2_	6.67 ± 0.28^a^	5.68 ± 0.49^a^	4.60 ± 0.45^b^	7.69 ± 0.70^a^	2.72 ± 0.79^a^	3.83 ± 0.48^a^	3.43 ± 0.36^c^	5.36 ± 0.44^abc^	4.08 ± 0.53b^c^	5.76 ± 0.67^ab^
	D_3_	4.43 ± 0.23^b^	5.73 ± 0.79^a^	5.03 ± 0.62^b^	3.74 ± 0.49^b^	1.78 ± 0.54^ab^	5.66 ± 0.90^a^	4.54 ± 0.56^bc^	5.08 ± 0.32^c^	5.09 ± 0.45^ab^	3.76 ± 0.30^c^
	D_1_	6.23 ± 0.52^a^	5.41 ± 0.90^a^	14.13 ± 6.27^a^	5.57 ± 1.04^b^	1.57 ± 0.21^ab^	5.71 ± 0.95^a^	3.50 ± 0.85^c^	3.37 ± 0.78^d^	4.19 ± 0.53^bc^	7.07 ± 0.33^a^
W_2_	D_2_	3.46 ± 0.44^b^	6.04 ± 0.97^a^	5.53 ± 0.83^ab^	4.35 ± 0.71^b^	1.26 ± 0.23^b^	5.82 ± 0.91^a^	4.67 ± 0.71^bc^	6.02 ± 0.23^ab^	5.35 ± 0.57^a^	3.61 ± 0.56^c^
	D_3_	4.11 ± 0.55^b^	5.07 ± 0.59^ab^	5.57 ± 1.00^ab^	3.85 ± 0.91^b^	2.33 ± 0.67^ab^	4.99 ± 0.53^a^	5.64 ± 0.81^ab^	6.20 ± 0.54^a^	4.85 ± 0.34^abc^	6.08 ± 0.34^a^
*p*-value		0.001	0.015	0.025	0.001	0.009	0.100	<0.001	<0.001	0.028	<0.001
Lsd		1.20	1.64	6.03	1.38	0.93	1.74	1.06	0.59	0.77	0.97
		**60 days after planting (2018)**					**60 days after planting (2019)**				
**Water level**	**Plant density**	**0–30 cm**	**30–60 cm**	**60–90 cm**	**90–120 cm**	**120–150 cm**	**0–30 cm**	**30–60 cm**	**60–90 cm**	**90–120 cm**	**120–150 cm**
	D_1_	2.88 ± 0.60	2.02 ± 0.25^c^	1.67 ± 0.49^b^	0.88 ± 0.17^c^	5.74 ± 1.20^ab^	7.22 ± 0.59	5.57 ± 0.52	5.67 ± 0.88^ab^	5.15 ± 0.62	5.32 ± 0.24^bc^
W_1_	D_2_	3.00 ± 0.54	3.95 ± 0.66^ab^	3.12 ± 0.93^a^	1.71 ± 0.55^bc^	7.69 ± 0.70^a^	7.37 ± 0.40	6.31 ± 0.51	4.77 ± 0.62^bc^	4.62 ± 0.64	5.36 ± 0.44^abc^
	D_3_	2.87 ± 0.60	2.74 ± 0.69^abc^	1.79 ± 0.54^ab^	1.23 ± 0.40^bc^	3.74 ± 0.49^b^	6.27 ± 0.32	6.06 ± 0.42	7.22 ± 0.40^a^	5.85 ± 0.40	5.08 ± 0.32^c^
	D_1_	2.22 ± 0.58	2.11 ± 0.46^bc^	1.73 ± 0.23^ab^	6.19 ± 1.36^a^	5.57 ± 1.04^b^	5.06 ± 1.10	6.22 ± 0.59	7.41 ± 0.38^a^	5.49 ± 1.23	3.37 ± 0.78^d^
W_2_	D_2_	4.37 ± 0.58	2.12 ± 0.38^bc^	1.26 ± 0.23^b^	6.56 ± 1.15^a^	4.35 ± 0.71^b^	3.27 ± 0.61	5.19 ± 1.01	3.50 ± 0.71^c^	5.75 ± 0.87	6.02 ± 0.23^ab^
	D_3_	2.72 ± 0.51	4.27 ± 1.04^a^	2.39 ± 0.70^ab^	2.57 ± 0.95^b^	3.85 ± 0.91^b^	5.67 ± 0.76	4.38 ± 1.03	6.23 ± 0.91^ab^	4.70 ± 0.50	6.20 ± 0.54^a^
*p*-value		0.127	0.002	0.002	<0.001	0.001	0.057	0.051	0.003	0.112	<0.001
Lsd		1.45	1.26	0.97	1.02	1.38	2.02	1.38	1.28	1.54	0.59
		**90 days after planting (2018)**					**90 days after planting (2019)**				
**Water level**	**Plant density**	**0–30 cm**	**30–60 cm**	**60–90 cm**	**90–120 cm**	**120–150 cm**	**0–30 cm**	**30–60 cm**	**60–90 cm**	**90–120 cm**	**120–150 cm**
	D_1_	2.85 ± 0.63	1.54 ± 0.28	1.43 ± 0.17	6.19 ± 1.23^a^	0.97 ± 0.21^d^	4.58 ± 0.87^bc^	5.83 ± 0.29	7.42 ± 0.49	5.86 ± 0.63^a^	4.77 ± 0.84
W_1_	D_2_	2.32 ± 0.60	1.75 ± 0.49	1.00 ± 0.22	6.46 ± 1.50^a^	1.92 ± 0.32^bc^	5.76 ± 0.67^ab^	2.55 ± 0.42	6.50 ± 0.99	4.59 ± 0.76^ab^	4.10 ± 0.80
	D_3_	1.59 ± 0.42	1.84 ± 0.46	1.12 ± 0.32	2.21 ± 0.33^c^	1.50 ± 0.30^cd^	3.76 ± 0.30^c^	2.86 ± 0.15	5.08 ± 0.60	5.88 ± 0.76^a^	5.30 ± 0.73
	D_1_	5.03 ± 0.80	3.73 ± 0.42	3.17 ± 0.74	3.67 ± 0.73^bc^	2.32 ± 0.20^ab^	7.07 ± 0.33^a^	5.78 ± 0.74	5.69 ± 0.75	3.94 ± 0.69^ab^	5.99 ± 0.47
W_2_	D_2_	3.88 ± 0.73	2.83 ± 0.34	2.10 ± 0.32	2.57 ± 0.60^bc^	2.28 ± 0.40^abc^	3.61 ± 0.56^c^	4.48 ± 0.50	4.57 ± 0.76	4.14 ± 0.84a^b^	5.65 ± 0.34
	D_3_	4.47 ± 0.73	2.84 ± 0.35	3.03 ± 0.83	3.74 ± 1.03^b^	2.72 ± 0.19^a^	6.08 ± 0.34^a^	5.12 ± 0.74	3.65 ± 0.21	2.52 ± 0.25^b^	4.86 ± 0.75
*p*-value		0.445	0.101	0.459	<0.001	0.024	<0.001	0.060	0.900	0.030	0.092
Lsd		1.46	0.87	0.97	1.00	0.53	0.97	1.45	1.55	1.50	1.35
		**120 days after planting (2018)**					**120 days after planting (2019)**				
**Water level**	**Plant density**	**0–30 cm**	**30–60 cm**	**60–90 cm**	**90–120 cm**	**120–150 cm**	**0–30 cm**	**30–60 cm**	**60–90 cm**	**90–120 cm**	**120–150 cm**
	D_1_	2.31 ± 0.48	2.28 ± 0.39^a^	1.45 ± 0.22	3.33 ± 1.13^a^	5.74 ± 1.20^ab^	4.95 ± 0.82	3.99 ± 0.63	3.77 ± 0.22^b^	2.89 ± 0.48^c^	4.58 ± 0.87^bc^
W_1_	D_2_	1.88 ± 0.46	1.32 ± 0.30^a^	0.83 ± 0.13	4.09 ± 1.08^a^	7.69 ± 0.70^a^	5.07 ± 0.69	6.48 ± 0.65	5.03 ± 0.64^ab^	4.29 ± 0.75^bc^	5.76 ± 0.67^ab^
	D_3_	1.65 ± 0.50	1.21 ± 0.29^a^	1.52 ± 0.47	1.12 ± 0.26^b^	3.74 ± 0.49^b^	5.48 ± 0.44	4.52 ± 0.55	4.43 ± 0.52^ab^	5.30 ± 1.11^ab^	3.76 ± 0.30^c^
	D_1_	2.64 ± 0.52	1.37 ± 0.40^a^	1.90 ± 0.42	1.34 ± 0.27^b^	5.57 ± 1.04^b^	4.70 ± 0.76	4.73 ± 0.53	3.97 ± 0.96^b^	3.56 ± 0.89^bc^	7.07 ± 0.33^a^
W_2_	D_2_	2.75 ± 0.45	2.18 ± 0.42^a^	2.38 ± 0.41	1.18 ± 0.26^b^	4.35 ± 0.71^b^	4.94 ± 0.85	6.39 ± 0.78	4.03 ± 0.67^b^	6.53 ± 0.53^a^	3.61 ± 0.56^c^
	D_3_	2.29 ± 0.48	1.95 ± 0.22^a^	2.14 ± 0.35	1.57 ± 0.29^b^	3.85 ± 0.91^b^	5.33 ± 0.84	4.30 ± 0.82	6.29 ± 0.60^a^	4.71 ± 0.76^abc^	6.08 ± 0.34^a^
*p*-value		0.735	0.002	0.080	<0.001	0.001	0.992	0.414	0.027	0.036	<0.001
Lsd		0.99	0.73	0.69	1.09	1.38	1.44	1.11	1.46	1.51	0.97
		**150 days after planting (2018)**					**150 days after planting (2019)**				
**Water level**	**Plant density**	**0–30 cm**	**30–60 cm**	**60–90 cm**	**90–120 cm**	**120–150 cm**	**0–30 cm**	**30–60 cm**	**60–90 cm**	**90–120 cm**	**120–150 cm**
	D_1_	3.63 ± 0.69	1.66 ± 0.32	0.97 ± 0.21^d^	1.67 ± 0.07	1.24 ± 0.18	6.91 ± 0.82	4.38 ± 0.63^bc^	4.77 ± 0.22	4.02 ± 0.48	5.86 ± 0.87^a^
W_1_	D_2_	3.83 ± 0.33	2.17 ± 0.34	1.92 ± 0.32^bc^	1.75 ± 0.13	1.06 ± 0.29	7.25 ± 0.69	6.18 ± 0.65^ab^	4.10 ± 0.64	2.91 ± 0.75	4.59 ± 0.67^ab^
	D_3_	4.48 ± 0.38	2.07 ± 0.43	1.50 ± 0.30^cd^	1.56 ± 0.13	1.22 ± 0.26	5.61 ± 0.44	4.42 ± 0.55^bc^	5.30 ± 0.52	2.90 ± 1.11	5.88 ± 0.30^a^
	D_1_	3.08 ± 0.43	2.63 ± 0.31	2.32 ± 0.20^ab^	1.23 ± 0.22	3.03 ± 0.64	7.48 ± 0.76	7.80 ± 0.53^a^	5.99 ± 0.96	5.12 ± 0.89	3.94 ± 0.33^ab^
W_2_	D_2_	3.27 ± 0.64	3.55 ± 0.44	2.28 ± 0.40^abc^	1.40 ± 0.15	2.11 ± 0.62	6.77 ± 0.85	4.20 ± 0.78^c^	5.65 ± 0.67	4.80 ± 0.53	4.14 ± 0.56^ab^
	D_3_	3.12 ± 0.32	2.53 ± 0.42	2.72 ± 0.29^a^	1.29 ± 0.33	3.51 ± 0.80	6.76 ± 0.84	6.03 ± 0.82^abc^	4.86 ± 0.60	3.76 ± 0.76	2.52 ± 0.34^b^
*p*-value		0.361	0.398	0.024	0.814	0.297	0.352	<0.001	0.092	0.254	0.030
Lsd		0.92	0.96	0.53	0.36	1.13	1.62	1.31	1.35	0.93	1.50

**Note:**

Irrigation (water level) comprised W_1_ = 5,250 m^3^/hm^2^ and W_2_ = 4,740 m^3^/hm^2^ and maize plant density included D_1_ = 75,000 plants/ha^−1^, D_2_ = 97,500 plants/ha^−1^, and D_3_ = 120,000 plants/ha. Values (mean ± standard error of mean) with different superscripts in the same column are significantly different at *p* < 0.05.

**Table 4:** Combined effect of nitrogen fertilizer rates and plant density on NH_3_–N emission across the 2 years. Nitrogen fertilizer consisted of N_0_ = 0 kg N ha^−1^, N_1_ = 270 kg N ha^−1^, and N_2_ = 360 kg N ha^−1^ and maize plant density included D_1_ = 75,000 plants/ha^−1^, D_2_ = 97,500 plants/ha^−1^, and D_3_ = 120,000 plants/ha^−1^. The error bars represent standard error of means (SEMs) from the triplicate readings. Means separation was done with least significant difference at *p*-value < 0.05. Means ± SEMs with a common superscript indicates no significant difference at *p* < 0.05 and those with otherwise are significantly different at *p* < 0.05.

N rate	Plant density	30 days after planting (2018)					30 days after planting (2019)				
		0–30 cm	30–60cm	60–90 cm	90–120 cm	120–150 cm	0–30 cm	30–60 cm	60–90 cm	90–120 cm	120–150 cm
	D_1_	6.31 ± 0.86^ab^	4.06 ± 1.32	3.40 ± 0.54	2.18 ± 0.60^d^	0.73 ± 0.17^b^	5.96 ± 0.93	4.64 ± 1.76^abc^	3.30 ± 1.26^d^	3.49 ± 0.65^b^	6.74 ± 0.38^a^
N_0_	D_2_	4.87 ± 0.75^abc^	5.52 ± 0.65	4.75 ± 0.71	4.14 ± 0.68^bcd^	1.12 ± 0.17^b^	6.30 ± 1.05	4.31 ± 0.96^abc^	6.10 ± 0.41^ab^	4.21 ± 0.32^b^	4.02 ± 1.19^b^
	D_3_	5.43 ± 0.98^abc^	4.13 ± 0.49	2.95 ± 0.65	3.09 ± 0.69^cd^	0.80 ± 0.15^b^	5.03 ± 0.73	4.83 ± 0.96^abc^	4.87 ± 0.49^c^	3.96 ± 0.22^b^	5.12 ± 0.55^ab^
	D_1_	5.58 ± 1.00^abc^	5.58 ± 0.77	17.28 ± 9.42	7.10 ± 1.21^a^	1.86 ± 0.20^ab^	5.75 ± 1.18	4.15 ± 0.64^bc^	4.47 ± 0.26^cd^	4.55 ± 0.11^b^	6.99 ± 0.37^a^
N_1_	D_2_	4.68 ± 1.08^bc^	6.90 ± 1.01	5.93 ± 0.59	6.39 ± 1.06^ab^	3.27 ± 0.15^a^	4.99 ± 0.95	4.70 ± 0.41^abc^	5.48 ± 0.32^bc^	4.95 ± 0.38^ab^	5.61 ± 0.85^ab^
	D_3_	4.12 ± 0.48^bc^	5.69 ± 0.90	7.03 ± 0.8	2.87 ± 0.62^cd^	2.07 ± 0.79^ab^	4.63 ± 0.66	4.87 ± 0.75^abc^	5.12 ± 0.23^bc^	4.76 ± 0.35^ab^	5.28 ± 0.84^ab^
	D_1_	7.19 ± 0.64^a^	2.45 ± 0.27	4.97 ± 1.02	7.68 ± 0.76^a^	1.93 ± 0.44^ab^	5.04 ± 0.58	6.40 ± 0.21^a^	5.26 ± 0.37^bc^	3.86 ± 0.68^b^	3.74 ± 1.19^b^
N_2_	D_2_	5.65 ± 0.61^ab^	5.18 ± 1.03	4.52 ± 1.08	7.53 ± 1.13^a^	1.59 ± 0.28^ab^	3.19 ± 0.38	3.14 ± 0.62^c^	5.50 ± 0.57^bc^	4.99 ± 1.17^ab^	4.44 ± 0.33^b^
	D_3_	3.25 ± 0.57^c^	6.38 ± 0.90	5.93 ± 0.74	5.43 ± 0.95^abc^	3.31 ± 0.72^a^	6.32 ± 1.18	5.57 ± 0.97^ab^	6.93 ± 0.60^a^	6.18 ± 0.30^a^	4.38 ± 0.45^b^
*p*-value		0.024	0.054	0.088	0.003	0.012	0.057	0.003	<0.001	0.021	0.002
Lsd		1.47	2.01	7.39	1.68	1.13	2.13	1.29	0.72	0.94	1.19
**N rate**	**Plant density**	**60 days after planting (2018)**					**60 days after planting (2019)**				
		**0–30 cm**	**30–60 cm**	**60–90 cm**	**90–120 cm**	**120–150 cm**	**0–30 cm**	**30–60 cm**	**60–90 cm**	**90–120 cm**	**120–150 cm**
	D_1_	1.85 ± 0.74	1.93 ± 0.38^b^	0.97 ± 0.36^c^	0.61 ± 0.09^c^	2.18 ± 0.60^d^	6.32 ± 1.14	7.48 ± 0.34	8.50 ± 0.37^a^	7.22 ± 1.24^a^	3.30 ± 1.26^d^
N_0_	D_2_	3.58 ± 0.36	2.90 ± 0.68^b^	1.14 ± 0.18^c^	1.65 ± 0.31^c^	4.14 ± 0.68^bcd^	5.39 ± 1.32	7.41 ± 0.52	5.31 ± 0.93^bc^	5.61 ± 0.99^ab^	6.10 ± 0.41^ab^
	D_3_	1.73 ± 0.22	1.23 ± 0.20^b^	0.78 ± 0.16^c^	0.68 ± 0.07^c^	3.09 ± 0.69^cd^	6.04 ± 0.72	6.90 ± 0.71	7.67 ± 0.60^ab^	5.03 ± 0.57^ab^	4.87 ± 0.49^c^
	D_1_	1.58 ± 0.17	1.80 ± 0.38^b^	1.92 ± 0.20^abc^	5.03 ± 1.77^ab^	7.10 ± 1.21^a^	4.89 ± 1.16	4.96 ± 0.50	5.66 ± 1.04^bc^	5.77 ± 1.00^ab^	4.47 ± 0.26^cd^
N_1_	D_2_	3.83 ± 0.95	2.48 ± 0.56^b^	3.80 ± 1.34^a^	6.18 ± 1.35^a^	6.39 ± 1.06^ab^	5.57 ± 0.93	3.51 ± 0.60	4.68 ± 0.25^cd^	4.22 ± 0.90^ab^	5.48 ± 0.32^bc^
	D_3_	3.46 ± 0.97	3.47 ± 0.85^ab^	2.07 ± 0.79^abc^	1.14 ± 0.22^c^	2.87 ± 0.62^cd^	6.26 ± 0.35	3.81 ± 1.31	4.95 ± 1.03^c^	4.87 ± 0.52^ab^	5.12 ± 0.23^bc^
	D_1_	4.23 ± 0.55	2.47 ± 0.56^b^	2.20 ± 0.63^abc^	4.98 ± 1.74^ab^	7.68 ± 0.76^a^	7.21 ± 1.10	5.25 ± 0.65	5.46 ± 0.53^bc^	2.97 ± 0.38^b^	5.26 ± 0.37^bc^
N_2_	D_2_	3.65 ± 0.88	3.73 ± 0.98^ab^	1.63 ± 0.26^bc^	4.56 ± 1.84^ab^	7.53 ± 1.13^a^	5.00 ± 1.09	6.32 ± 1.00	2.41 ± 0.73^d^	5.72 ± 0.95^ab^	5.50 ± 0.57^bc^
	D_3_	3.20 ± 0.39	5.83 ± 1.10^a^	3.43 ± 0.76^ab^	3.88 ± 1.25^b^	5.43 ± 0.95^abc^	5.61 ± 1.00	4.94 ± 0.44	7.56 ± 0.40^ab^	5.93 ± 0.67^ab^	6.93 ± 0.60^a^
*p*-value		0.084	0.004	0.002	<0.001	0.003	0.438	0.330	0.001	0.002	<0.001
Lsd		1.78	1.54	1.19	1.25	1.68	2.47	1.69	1.57	1.88	0.72
**N rate**	**Plant density**	**90 days after planting (2018)**					**90 days after planting (2019)**				
		**0–30 cm**	**30–60 cm**	**60–90 cm**	**90–120 cm**	**120–150 cm**	**0–30 cm**	**30–60 cm**	**60–90 cm**	**90–120 cm**	**120–150 cm**
	D_1_	3.05 ± 0.64	1.80 ± 0.43	1.40 ± 0.31	2.90 ± 0.81^de^	1.93 ± 0.24^ab^	6.74 ± 0.38^a^	7.00 ± 0.60	6.77 ± 0.45^ab^	6.84 ± 1.19^a^	4.60 ± 0.49
N_0_	D_2_	1.58 ± 0.44	1.40 ± 0.42	0.93 ± 0.32	1.03 ± 0.32^ef^	1.83 ± 0.20^ab^	4.02 ± 1.19^b^	3.91 ± 0.77	7.08 ± 1.37^a^	6.39 ± 1.85^ab^	4.58 ± 0.78
	D_3_	1.48 ± 0.23	1.73 ± 0.50	0.95 ± 0.28	0.85 ± 0.23^f^	1.50 ± 0.31^b^	5.12 ± 0.55^ab^	3.92 ± 1.21	3.80 ± 0.42^b^	3.94 ± 0.12^abc^	3.38 ± 0.43
	D_1_	4.33 ± 0.70	2.83 ± 0.84	1.75 ± 0.37	4.96 ± 1.66^bc^	1.13 ± 0.34^b^	6.99 ± 0.37^a^	5.38 ± 0.74	7.53 ± 0.59^a^	4.57 ± 0.93^abc^	4.57 ± 0.73
N_1_	D_2_	3.53 ± 0.70	2.33 ± 0.68	2.13 ± 0.48	5.28 ± 1.80^abc^	1.75 ± 0.54^ab^	5.61 ± 0.85^ab^	3.37 ± 0.58	4.45 ± 0.62^ab^	3.57 ± 0.63^bc^	4.17 ± 0.91
	D_3_	4.08 ± 0.80	2.55 ± 0.43	2.33 ± 0.83	3.48 ± 0.94^cd^	2.68 ± 0.15^a^	5.28 ± 0.84^ab^	4.40 ± 0.71	4.76 ± 0.46^ab^	4.30 ± 0.08^abc^	5.53 ± 0.68
	D_1_	4.45 ± 1.43	3.28 ± 0.48	3.75 ± 0.97	6.93 ± 0.91^ab^	1.88 ± 0.48^ab^	3.74 ± 1.19^b^	5.03 ± 0.42	5.37 ± 1.17^ab^	3.30 ± 0.02^c^	6.97 ± 0.95
N_2_	D_2_	4.20 ± 1.04	3.15 ± 0.28	1.60 ± 0.25	7.23 ± 1.08^a^	2.73 ± 0.45^a^	4.44 ± 0.33^b^	3.27 ± 0.79	5.08 ± 1.14^ab^	3.12 ± 0.31^c^	5.87 ± 0.65
	D_3_	3.53 ± 1.22	2.75 ± 0.63	2.95 ± 1.12	4.60 ± 0.84^cd^	2.15 ± 0.52^ab^	4.38 ± 0.45^b^	3.64 ± 0.35	4.53 ± 0.89^ab^	4.34 ± 0.48^abc^	6.34 ± 1.04
*p*-value		0.726	0.879	0.051	0.028	0.001	0.002	0.491	0.028	0.020	0.190
Lsd		1.79	1.06	1.19	1.22	0.65	1.19	1.78	1.90	1.83	1.66
**N rate**	**Plant density**	**120 days after planting (2018)**					**120 days after planting (2019)**				
		**0–30 cm**	**30–60 cm**	**60–90 cm**	**90–120 cm**	**120–150 cm**	**0–30 cm**	**30–60 cm**	**60–90 cm**	**90–120 cm**	**120–150 cm**
	D_1_	0.71 ± 0.13	1.16 ± 0.25^b^	1.13 ± 0.31^bc^	1.23 ± 0.23^b^	2.18 ± 0.60^d^	4.96 ± 0.74	4.29 ± 0.44	4.11 ± 0.90^ab^	4.91 ± 0.98^ab^	6.74 ± 0.38^a^
N_0_	D_2_	1.38 ± 0.23	0.65 ± 0.22^b^	0.90 ± 0.23^c^	1.05 ± 0.18^b^	4.14 ± 0.68^bcd^	5.51 ± 0.55	5.64 ± 0.16	5.21 ± 0.73^ab^	5.73 ± 0.69^a^	4.02 ± 1.19^b^
	D_3_	1.20 ± 0.23	0.93 ± 0.20^b^	1.81 ± 0.20^abc^	1.33 ± 0.21^b^	3.09 ± 0.69^cd^	5.94 ± 0.39	4.61 ± 0.57	4.25 ± 0.62^ab^	6.14 ± 0.66^a^	5.12 ± 0.55^ab^
	D_1_	3.02 ± 0.44	2.83 ± 0.69^a^	2.80 ± 0.29^a^	4.39 ± 1.55^a^	7.10 ± 1.21^a^	2.62 ± 0.59	4.66 ± 1.11	4.70 ± 0.74^ab^	2.66 ± 0.37^b^	6.99 ± 0.37^a^
N_1_	D_2_	2.10 ± 0.55	1.73 ± 0.39^ab^	1.63 ± 0.43^abc^	4.42 ± 1.72^a^	6.39 ± 1.06^ab^	4.25 ± 1.11	6.55 ± 1.18	2.66 ± 0.58^b^	5.90 ± 1.17^a^	5.61 ± 0.85^ab^
	D_3_	1.58 ± 0.37	1.99 ± 0.42^ab^	0.76 ± 0.10^c^	1.43 ± 0.32^b^	2.87 ± 0.62^cd^	4.31 ± 0.99	3.63 ± 0.82	5.52 ± 0.83^ab^	2.61 ± 0.94^b^	5.28 ± 0.84^ab^
	D_1_	3.70 ± 0.21	1.50 ± 0.30^ab^	1.10 ± 0.15^bc^	1.38 ± 0.33^b^	7.68 ± 0.76^a^	6.89 ± 0.53	4.13 ± 0.51	2.80 ± 0.77^b^	2.10 ± 0.73^b^	3.74 ± 1.19^b^
N_2_	D_2_	3.48 ± 0.55	2.88 ± 0.23^a^	2.37 ± 0.57^ab^	2.45 ± 0.28^ab^	7.53 ± 1.13^a^	5.26 ± 1.06	7.12 ± 0.91	5.71 ± 0.52^a^	4.61 ± 0.84^ab^	4.44 ± 0.33^b^
	D_3_	3.14 ± 0.78	1.83 ± 0.22^ab^	2.93 ± 0.61^a^	1.28 ± 0.48^b^	5.43 ± 0.95^abc^	5.96 ± 0.82	4.98 ± 1.07	6.31 ± 0.73^a^	6.26 ± 1.16^a^	4.38 ± 0.45^b^
*p*-value		0.223	0.005	<0.001	0.004	0.003	0.105	0.132	0.001	0.002	0.002
Lsd		1.21	0.89	0.85	1.34	1.68	1.77	1.36	1.79	1.84	1.19
**N rate**	**Plant density**	**150 days after planting (2018)**					**150 days after planting (2019)**				
		**0–30 cm**	**30–60cm**	**60–90cm**	**90–120cm**	**120–150 cm**	**0–30 cm**	**30–60 cm**	**60–90 cm**	**90–120 cm**	**120–150 cm**
	D_1_	1.70 ± 0.43^d^	1.33 ± 0.38	1.93 ± 0.24^ab^	1.73 ± 0.09	1.17 ± 0.30	7.50 ± 0.74	5.80 ± 0.44^abc^	4.60 ± 0.90	4.06 ± 0.98^b^	6.84 ± 0.38^a^
N_0_	D_2_	2.70±±0.35^cd^	2.48 ± 0.60	1.83 ± 0.20^ab^	1.73 ± 0.09	0.73 ± 0.33	7.90 ± 0.55	7.09 ± 0.16^ab^	4.58 ± 0.73	3.15 ± 0.69^b^	6.39 ± 1.19^ab^
	D_3_	3.25 ± 0.72^abcd^	1.80 ± 0.27	1.50 ± 0.31^b^	1.48 ± 0.31	1.17 ± 0.23	6.40 ± 0.39	7.92 ± 0.57^a^	3.38 ± 0.62	3.30 ± 0.66^b^	3.94 ± 0.55^abc^
	D_1_	3.58 ± 0.89^abc^	2.26 ± 0.41	1.13 ± 0.34^b^	1.03 ± 0.29	2.15 ± 0.41	7.51 ± 0.59	6.86 ± 1.11^ab^	4.57 ± 0.74	3.59 ± 0.37^b^	4.57 ± 0.38^abc^
N_1_	D_2_	4.93 ± 0.09^a^	2.68 ± 0.53	1.75 ± 0.54^ab^	1.13 ± 0.18	1.86 ± 0.47	6.21 ± 1.11	5.01 ± 1.18^bcd^	4.17 ± 0.58	4.90 ± 1.17^ab^	3.57 ± 0.85^bc^
	D_3_	3.90 ± 0.64^abc^	2.46 ± 0.60	2.68 ± 0.15^a^	0.84 ± 0.28	2.95 ± 0.96	6.64 ± 0.99	4.66 ± 0.82^bcd^	5.53 ± 0.83	3.51 ± 0.94^b^	4.30 ± 0.84^abc^
	D_1_	4.80 ± 0.26^ab^	2.85 ± 0.28	1.88 ± 0.48^ab^	1.60 ± 0.06	3.09 ± 0.95	6.57 ± 0.53	5.62 ± 0.51^abcd^	6.97 ± 0.77	6.06 ± 0.73^a^	3.30 ± 1.19^c^
N_2_	D_2_	3.03 ± 0.38^bcd^	3.43 ± 0.53	2.73 ± 0.45^a^	1.88 ± 0.12	2.16 ± 0.68	6.92 ± 1.06	3.47 ± 0.91^cd^	5.87 ± 0.52	3.53 ± 0.84^b^	3.12 ± 0.33^c^
	D_3_	4.25 ± 0.00^abc^	2.65 ± 0.62	2.15 ± 0.52^ab^	1.94 ± 0.11	2.98 ± 1.04	5.52 ± 0.82	3.11 ± 1.08^d^	6.34 ± 0.73	3.19 ± 1.16^b^	4.34 ± 0.45^abc^
*p*-value		0.001	0.844	0.001	0.320	0.851	0.569	0.001	0.190	<0.001	0.020
Lsd		1.13	1.18	0.65	0.44	1.38	1.99	1.61	1.66	1.13	1.83

**Note:**

Nitrogen fertilizer consisted of N_0_ = 0 kg N ha^−1^, N_1_ = 270 kg N ha^−1^, and N_2_ = 360 kg N ha^−1^ and maize plant density included D_1_ = 75,000 plants/ha^−1^, D_2_ = 97,500 plants/ha^−1^, and D_3_ = 120,000 plants/ha^−1^. Values (mean ± standard error of mean) with different superscripts in the same column are significantly different at *p* < 0.05.

**Table 5:** Combined effect of N fertilizer rates and plant density on NO_3_–N leaching and accumulation on maize farmland. Irrigation (water level) comprised of W_1_ = 5,250 m^3^/hm^2^ and W_2_ = 4,740 m^3^/hm^2^, nitrogen fertilizer consisted of N_0_ = 0 kg N ha^−1^, N_1_ = 270 kg N ha^−1^, and N_2_ = 360 kg N ha^−1^ and maize plant density included D_1_ = 75,000 plants/ha^−1^, D_2_ = 97,500 plants/ha^−1^, and D_3_ = 120,000 plants/ha^−1^. Means separation was done with least significant difference at *p* < 0.05. Means ± SEMs with a common superscript indicates no significant difference at *p* < 0.05 and those with otherwise are significantly different at *p* < 0.05.

Water level	N rate	Plant density	30 days after planting and soil depth (2018)					30 days after planting and soil depth (2019)				
			0–30 cm	30–60cm	60–90cm	90–120cm	120–150 cm	0–30 cm	30–60 cm	60–90 cm	90–120 cm	120–150 cm
		D_1_	7.65 ± 0.89^abc^	2.05 ± 0.03	3.20 ± 0.46^b^	1.50 ± 0.69^f^	0.43 ± 0.07^c^	6.03 ± 0.24^ab^	8.57 ± 0.29^a^	6.12 ± 0.01^bcd^	4.40 ± 0.25^bcd^	6.29 ± 0.20^abcd^
	N_0_	D_2_	6.40 ± 0.06^abcd^	5.20 ± 0.35	5.40 ± 0.81^b^	5.25 ± 1.01^bcdef^	1.400.15^c^	4.34 ± 0.94^ab^	2.22 ± 0.16^def^	7.02 ± 0.11^ab^	4.17 ± 0.49^cd^	6.40 ± 0.15^abcd^
		D_3_	6.90 ± 0.98^abcd^	4.50 ± 1.04	3.90 ± 1.10^b^	3.65 ± 1.36^def^	1.00 ± 0.06^c^	3.40 ± 0.11^ab^	2.71 ± 0.14^def^	4.71 ± 0.94^de^	4.42 ± 0.16^bcd^	4.30 ± 0.89^bcde^
		D_1_	3.35 ± 0.14^de^	3.90 ± 0.40	2.40 ± 0.00^b^	9.67 ± 0.23^a^	1.75 ± 0.23^bc^	6.46 ± 0.53^ab^	5.03 ± 1.09^bcde^	5.05 ± 0.01^cde^	4.42 ± 0.21^bcd^	6.36 ± 0.15^abcd^
W_1_	N_1_	D_2_	6.80 ± 0.92^abcd^	4.70 ± 0.31	4.90 ± 0.75^b^	8.73 ± 0.23^abc^	5.73 ± 0.73^a^	4.55 ± 0.63^ab^	3.81 ± 0.12^bcdef^	4.79 ± 0.09^de^	5.47 ± 0.55^abc^	6.86 ± 1.33^abc^
		D_3_	3.83 ± 0.33^cde^	5.93 ± 1.99	5.50 ± 0.75^b^	3.77 ± 0.86^def^	2.43b±1.70^c^	4.64 ± 1.11^ab^	6.27 ± 0.72^abc^	4.82 ± 0.27^de^	4.04 ± 0.23^cd^	3.49 ± 0.29^def^
		D_1_	8.45 ± 0.55^a^	2.00 ± 0.29	3.30 ± 0.69^b^	6.05 ± 0.09^abcde^	2.15 ± 0.92^bc^	3.89 ± 0.51^ab^	6.27 ± 0.40^abc^	4.79 ± 0.44^de^	2.40 ± 0.21^d^	1.10 ± 0.03^f^
	N_2_	D_2_	6.80 ± 0.17^abcd^	7.15 ± 1.01	3.50 ± 0.46^b^	9.10 ± 0.46^ab^	1.03 ± 0.23^c^	2.62 ± 0.63^b^	4.26 ± 0.61^bcde^	4.29 ± 0.39^de^	2.62 ± 0.88^d^	4.03 ± 0.03^cdef^
		D_3_	2.55 ± 0.49^e^	6.75 ± 1.07	5.70 ± 1.39^b^	3.80 ± 0.58^def^	1.92 ± 0.19^bc^	8.94 ± 0.10^a^	4.64 ± 0.02^bcde^	5.70 ± 0.12^bcde^	6.81 ± 023^ab^	3.51 ± 0.13^def^
		D_1_	4.97 ± 1.03^abcde^	6.07 ± 2.15	3.60 ± 1.10^b^	2.87 ± 0.92^def^	1.03 ± 0.23^c^	5.88 ± 2.06^ab^	0.71 ± 0.02^f^	0.48 ± 0.04^f^	2.59 ± 1.12^d^	7.19 ± 0.70^ab^
	N_0_	D_2_	3.33 ± 0.67^de^	5.83 ± 1.37	4.10 ± 1.21^b^	3.03 ± 0.20^def^	0.83 ± 0.19^c^	8.27 ± 0.87^a^	6.40 ± 0.49^abc^	5.18 ± 0.01^bcde^	4.25 ± 0.52^cd^	1.63 ± 0.29^ef^
		D_3_	3.97 ± 1.29^bcde^	3.75 ± 0.03	2.00 ± 0.00^b^	2.53 ± 0.46^ef^	0.60 ± 0.26^c^	6.67 ± 0.08^ab^	6.96 ± 0.18^ab^	5.04 ± 0.55^cde^	3.51 ± 0.05^cd^	5.94 ± 0.25^abcd^
		D_1_	7.80 ± 0.00^ab^	7.25 ± 0.14	32.15 ± 14.92^a^	4.53 ± 0.81^cdef^	1.97 ± 0.38^bc^	5.04 ± 2.49^ab^	3.26 ± 0.27^cdef^	3.89 ± 0.00^e^	4.67 ± 0.04^bcd^	7.62 ± 0.53^a^
W_2_	N_1_	D_2_	2.55 ± 0.66^e^	9.10 ± 0.40	6.95 ± 0.32^b^	4.05 ± 0.26^def^	0.80 ± 0.06^c^	5.43 ± 1.97^ab^	5.59 ± 0.14^abcd^	6.17 ± 0.14^bcd^	4.43 ± 0.41^bcd^	4.36 ± 0.53^bcde^
		D_3_	4.40 ± 0.98^bcde^	5.45 ± 0.14	8.55 ± 0.55^b^	1.97 ± 59^ef^	1.70 ± 0.35^c^	4.62 ± 0.97^ab^	3.46 ± 0.59^cdef^	5.42 ± 0.32^bcde^	5.48 ± 0.23^abc^	7.06 ± 0.50^abc^
		D_1_	5.93 ± 0.38^abcde^	2.90 ± 0.29	6.63 ± 1.40^b^	9.30 ± 0.50^ab^	1.72 ± 0.32^c^	6.20 ± 0.29^ab^	6.53 ± 0.19^abc^	5.73 ± 0.52^bcde^	5.32 ± 0.35^abc^	6.39 ± 0.35^abcd^
	N_2_	D_2_	4.50 ± 0.69^bcde^	3.20 ± 0.64	5.53 ± 2.14^b^	5.97 ± 1.92^abcde^	2.15 ± 0.14^bc^	3.75 ± 0.08^ab^	2.02 ± 0.54^ef^	6.72 ± 0.12^abc^	7.36 ± 0.64^a^	4.84 ± 0.61^abcd^
		D_3_	3.95 ± 0.95^cde^	6.00 ± 1.67	6.17 ± 0.89^b^	7.05 ± 1.24^abcd^	4.70 ± 0.81^ab^	3.70 ± 0.33^ab^	6.50 ± 1.97^abc^	8.16 ± 0.51^a^	5.56 ± 0.09^abc^	5.25 ± 0.47^abcd^
*p*-value			<0.001	0.067	0.048	0.020	0.001	0.010	<0.001	<0.001	<0.001	0.004
Lsd			2.07	2.84	10.45	2.38	1.60	3.01	1.83	1.02	1.33	1.68
**Water level **	**N rate**	**Plant density**	**60 days after planting and soil depth (2018)**					**60 days after planting and soil depth (2019)**				
			**0–30 cm**	**30–60 cm**	**60–90 cm**	**90–120 cm**	**120–150 cm**	**0–30 cm**	**30–60 cm**	**60–90 cm**	**90–120 cm**	**120–150 cm**
		D_1_	3.10 ± 1.10	2.55 ± 0.09^bc^	0.50 ± 0.06^c^	0.45 ± 0.09^d^	1.50 ± 0.69^f^	7.32 ± 0.98	7.15 ± 0.64^a^	8.83 ± 0.64^a^	4.50 ± 0.57^bc^	6.12 ± 0.01^bcd^
	N_0_	D_2_	3.05 ± 0.20	4.40 ± 0.17^abc^	1.45 ± 0.20^c^	1.30 ± 0.52^d^	5.25 ± 1.01^bcdef^	8.31 ± 0.19	6.89 ± 0.46^a^	6.00 ± 0.77^abcde^	4.25 ± 0.64^bc^	7.02 ± 0.11^ab^
		D_3_	2.05 ± 0.38	0.95 ± 0.32^c^	1.00 ± 0.06^c^	0.75 ± 0.09^d^	3.65 ± 1.36^def^	6.89 ± 0.90	6.11 ± 0.88	7.55 ± 1.24^abc^	6.14 ± 0.24^abc^	4.71 ± 0.94^de^
		D_1_	1.45 ± 0.09	2.20 ± 0.40^bc^	1.85 ± 0.20^bc^	1.10 ± 0.40^d^	9.67 ± 0.23^a^	5.75 ± 0.84	4.74 ± 0.22	3.54 ± 0.58^cdef^	7.30 ± 0.54^ab^	5.05 ± 0.09^cde^
W_1_	N_1_	D_2_	4.10 ± 1.44	2.25 ± 0.55^bc^	6.80 ± 0.17^a^	3.37 ± 1.03^cd^	8.73 ± 0.23^abc^	6.49 ± 0.79	4.85 ± 0.01	5.13 ± 0.02^abcdef^	5.27 ± 1.69^abc^	4.79 ± 0.09^de^
		D_3_	3.37 ± 1.92	3.63 ± 1.41^bc^	2.43 ± 1.70^bc^	0.83 ± 0.39^d^	3.77 ± 0.86^def^	5.76 ± 0.12	6.56 ± 0.94	7.20 ± 0.10^abcd^	5.55 ± 0.19^abc^	4.82 ± 0.27^de^
		D_1_	4.10 ± 1.15	1.30 ± 0.40^bc^	2.65 ± 1.30^bc^	1.10 ± 0.17^d^	6.05 ± 0.09^abcde^	8.60 ± 0.63	4.82 ± 0.96	4.66 ± 0.82^bcdef^	3.64 ± 0.54^bc^	4.79 ± 0.44^de^
	N_2_	D_2_	1.85 ± 0.43	5.20 ± 1.62^ab^	1.10 ± 0.17^c^	0.45 ± 0.14^d^	9.10 ± 0.46^ab^	7.31 ± 0.64	7.19 ± 1.15	3.17 ± 1.40^def^	4.35 ± 1.16^bc^	4.29 ± 0.39^de^
		D_3_	3.20 ± 0.06	3.65 ± 1.13^bc^	1.95 ± 0.20^bc^	2.10 ± 1.10^d^	3.80 ± 0.58^def^	6.12 ± 0.29	5.52 ± 0.41	6.92 ± 0.56^abcd^	5.86 ± 1.32^abc^	5.70 ± 0.12^bcde^
		D_1_	0.60 ± 0.06	1.30 ± 0.58^bc^	1.43 ± 0.53^c^	0.77 ± 0.07^d^	2.87 ± 0.92^def^	5.33 ± 2.14	7.81 ± 0.22	8.17 ± 0.39^ab^	9.94 ± 0.03^a^	0.48 ± 0.04^f^
	N_0_	D_2_	4.10 ± 0.58	1.40 ± 0.17^bc^	0.83 ± 0.19^c^	2.00 ± 0.29^d^	3.03 ± 0.20^def^	2.47 ± 0.02	7.93 ± 0.93	4.62 ± 1.81^bcdef^	6.97 ± 1.63^abc^	5.18 ± 0.01^bcde^
		D_3_	1.40 ± 0.06	1.50 ± 0.17^bc^	0.57 ± 0.29^c^	0.60 ± 0.12^d^	2.53 ± 0.46^ef^	5.18 ± 1.03	7.70 ± 1.06	7.78 ± 0.53^ab^	3.92 ± 0.58^bc^	5.04 ± 0.55^cde^
		D_1_	1.70 ± 0.35	1.40 ± 0.64^bc^	2.00 ± 0.40^bc^	8.95 ± 0.38^a^	4.53 ± 0.81^cdef^	4.04 ± 2.31	5.17 ± 1.07	7.79 ± 0.73^ab^	4.24 ± 1.54^bc^	3.89 ± 0.00^e^
W_2_	N_1_	D_2_	3.55 ± 1.53	2.70 ± 1.10^bc^	0.80 ± 0.06^c^	9.00 ± 0.23^a^	4.05 ± 0.26^def^	4.66 ± 1.68	2.18 ± 0.21	4.23 ± 0.32^bcdef^	3.18 ± 0.29^bc^	6.17 ± 0.14^bcd^
		D_3_	3.55 ± 1.01	3.30 ± 1.27^bc^	1.70 ± 0.35^c^	1.45 ± 0.03^d^	1.97 ± 0.59^ef^	6.75 ± 0.59	1.07 ± 0.30	2.71 ± 0.53^ef^	4.20 ± 0.94^bc^	5.42 ± 0.32^bcde^
		D_1_	4.35 ± 0.38	3.63 ± 0.23^bc^	1.75 ± 0.32^c^	8.87 ± 0.30^ab^	9.30 ± 0.50^ab^	5.83 ± 1.94	5.69 ± 1.00	6.27 ± 0.28^abcde^	2.30 ± 0.01^c^	5.73 ± 0.52^bcde^
	N_2_	D_2_	5.45 ± 0.66	2.25 ± 0.14^bc^	2.15 ± 0.14^bc^	8.67 ± 0.12^ab^	5.97 ± 1.92^abcde^	2.69 ± 0.42	5.46 ± 1.71	1.65 ± 0.31^f^	7.10 ± 1.14^abc^	6.72 ± 0.12^abc^
		D_3_	3.20 ± 0.87	8.00 ± 0.33^a^	4.90 ± 0.81^ab^	5.65 ± 1.88^bc^	7.05 ± 1.24^abcd^	5.07 ± 2.16	4.37 ± 0.70	8.19 ± 0.30^ab^	5.99 ± 0.72^abc^	8.16 ± 0.51^a^
*p*-value			0.284	0.009	<0.001	0.007	0.020	0.874	0.077	0.001	0.004	<0.001
Lsd			2.51	2.18	1.69	1.77	2.38	3.50	2.38	2.21	2.66	1.02
**Water level **	**N rate**	**Plant density**	**90 days after planting and soil depth (2018)**					**90 days after planting and soil depth (2019)**				
			**0–30 cm**	**30–60 cm**	**60–90 cm**	**90–120 cm**	**120–150 cm**	**0–30 cm**	**30–60 cm**	**60–90 cm**	**90–120 cm**	**120–150 cm**
		D_1_	3.40 ± 1.21	1.05 ± 0.32	1.20 ± 0.40^b^	1.30 ± 0.29^ef^	1.55 ± 0.38^cdef^	6.29 ± 0.20^abcd^	6.12 ± 0.94	6.69 ± 0.92^abc^	7.64 ± 0.11	3.52 ± 0.24
	N_0_	D_2_	0.65 ± 0.32	0.65 ± 0.03	0.45 ± 0.14^b^	0.50 ± 0.06^f^	1.50 ± 0.23^cdef^	6.40 ± 1.15^abcd^	3.87 ± 0.72	9.15 ± 0.28^a^	7.11 ± 0.27	2.90 ± 0.25
		D_3_	1.00 ± 0.06	0.80 ± 0.31	1.10 ± 0.58^b^	1.35 ± 0.03^def^	0.85 ± 0.26^ef^	4.30 ± 0.89^bcde^	2.57 ± 0.07	3.70 ± 0.89^bc^	5.58 ± 2.10	3.22 ± 0.21
		D_1_	3.80 ± 1.21	1.00 ± 0.06	1.50 ± 0.23^b^	8.67 ± 0.18^a^	0.45 ± 0.03^f^	6.36 ± 0.15^abcd^	5.69 ± 0.16	7.68 ± 1.11^abc^	6.05 ± 0.43	3.18 ± 0.73
W_1_	N_1_	D_2_	3.25 ± 1.07	0.90 ± 0.12	1.20 ± 0.52^b^	9.27 ± 0.58^a^	2.50 ± 0.92^abcde^	6.86 ± 1.33^abc^	2.26 ± 0.30	3.51 ± 0.77^bc^	2.09 ± 0.63	2.22 ± 0.47
		D_3_	2.77 ± 0.97	2.03 ± 0.44	1.67 ± 0.78^b^	2.53 ± 0.84^cdef^	2.60 ± 0.21^abcd^	3.49 ± 0.29^def^	3.07 ± 0.34	5.48 ± 0.70^abc^	5.29 ± 1.37	4.91 ± 0.90
		D_1_	1.35 ± 0.26	2.57 ± 0.27	1.60 ± 0.29^b^	8.60 ± 0.29^a^	0.90 ± 0.29^ef^	1.10 ± 0.03^f^	5.68 ± 0.28	7.89 ± 0.66^ab^	3.91 ± 1.01	7.61 ± 1.34
	N_2_	D_2_	3.05 ± 1.01	3.70 ± 0.00	1.35 ± 0.26^b^	9.60 ± 0.06^a^	1.75 ± 0.26^bcdef^	4.03 ± 0.03^cdef^	1.51 ± 0.18	6.84 ± 1.76^abc^	4.57 ± 0.50	7.18 ± 0.39
		D_3_	1.00 ± 0.29	2.70 ± 1.15	0.60 ± 0.12^b^	2.75 ± 0.26^cdef^	1.05 ± 0.26^def^	3.51 ± 0.13^def^	2.94 ± 0.31	6.05 ± 1.25^abc^	6.76 ± 0.10	7.78 ± 0.51
		D_1_	2.70 ± 0.69	2.55 ± 0.49	1.60 ± 0.52^b^	4.50 ± 0.81^bcd^	2.30 ± 0.06^abcde^	7.19 ± 0.70^ab^	7.89 ± 0.37	6.85 ± 0.41^abc^	6.05 ± 1.19	5.68 ± 0.84
	N_0_	D_2_	2.50 ± 0.17	2.15 ± 0.55	1.40 ± 0.52^b^	1.55 ± 0.49^def^	2.15 ± 0.20^abcde^	1.63 ± 0.29^ef^	3.95 ± 1.56	5.00 ± 2.25^abc^	5.68 ± 1.85	6.26 ± 0.80
		D_3_	1.95 ± 0.20	2.65 ± 0.55	0.80 ± 0.17^b^	0.35 ± 0.09^f^	2.15 ± 0.03^abcde^	5.94 ± 0.25^abcd^	5.27 ± 2.34	3.90 ± 0.28^bc^	2.31 ± 0.12	3.54 ± 0.73
		D_1_	4.85 ± 0.84	4.65 ± 0.43	2.00 ± 0.75^b^	1.25 ± 0.09^ef^	1.80 ± 0.35^bcdef^	7.62 ± 0.53^a^	5.07 ± 1.61	7.38 ± 0.70^abc^	3.09 ± 0.93	5.96 ± 0.05
W_2_	N_1_	D_2_	3.80 ± 1.10	3.75 ± 0.55	3.05 ± 0.20^ab^	1.30 ± 0.23^ef^	1.00 ± 0.23^def^	4.36 ± 0.53^bcde^	4.47 ± 0.59	5.39 ± 0.65^abc^	5.06 ± 0.63	6.12 ± 0.36
		D_3_	5.40 ± 0.75	3.07 ± 0.70	3.00 ± 1.55^ab^	4.43 ± 1.68^bcde^	2.75 ± 0.26^abc^	7.06 ± 0.50^abc^	5.73 ± 81	4.03 ± 0.17^bc^	3.32 ± 0.08	6.15 ± 0.92
		D_1_	7.55 ± 0.78	4.00 ± 0.75	5.90 ± 0.17^a^	5.25 ± 1.13^bc^	2.85 ± 0.32^abc^	6.39 ± 0.35^abcd^	4.39 ± 0.63	2.85 ± 0.19^c^	2.69 ± 0.02	6.34 ± 0.43
	N_2_	D_2_	5.35 ± 1.76	2.60 ± 0.29	1.85 ± 0.43^b^	4.85 ± 0.38^bc^	3.70 ± 0.12^a^	4.84 ± 0.61^abcd^	5.02 ± 0.01	3.33 ± 0.57^bc^	1.68 ± 0.31	4.57 ± 0.36
		D_3_	6.05 ± 1.01	2.80 ± 0.81	5.30 ± 0.87^a^	6.45 ± 0.14^ab^	3.25 ± 0.26^ab^	5.25 ± 0.47^abcd^	4.35 ± 0.02	3.01 ± 0.32^bc^	1.92 ± 0.48	4.91 ± 1.05
*p*-value			0.153	0.128	0.007	<0.001	0.022	0.004	0.120	0.038	0.061	0.705
Lsd			2.53	1.50	1.68	1.73	0.92	1.68	2.52	2.69	2.59	2.34
**Water level **	**N rate**	**Plant density**	**120 days after planting and soil depth (2018)**					**120 days after planting and soil depth (2019)**				
			**0–30 cm**	**30–60 cm**	**60–90 cm**	**90–120 cm**	**120–150 cm**	**0–30 cm**	**30–60 cm**	**60–90 cm**	**90–120 cm**	**120–150 cm**
		D_1_	0.45 ± 0.03	1.25 ± 0.32	0.95 ± 0.20	1.50 ± 0.12^b^	1.50 ± 0.69^f^	3.64 ± 0.81^abc^	4.74 ± 0.25^bcd^	3.08 ± 0.18^abc^	2.95 ± 0.54^bcd^	6.29 ± 0.20^abcd^
	N_0_	D_2_	1.60 ± 0.29	0.40 ± 0.00	0.55 ± 0.09	1.45 ± 0.03^b^	5.25 ± 1.01^bcdef^	4.32 ± 0.13^abc^	5.28 ± 0.05^bcd^	6.54 ± 0.88^ab^	6.07 ± 1.08^abcd^	6.40 ± 0.15^abcd^
		D_3_	0.80 ± 0.29	0.65 ± 0.26	1.45 ± 0.14	1.35 ± 0.09^b^	3.65 ± 1.36^def^	5.76 ± 0.71^abc^	5.48 ± 0.87^bc^	3.46 ± 1.05^abc^	5.86 ± 0.23^abcd^	4.30 ± 0.89^bcde^
		D_1_	3.13 ± 0.32	3.50 ± 061	2.25 ± 0.09	7.13 ± 2.06^a^	9.67 ± 0.23^a^	3.38 ± 1.05^abc^	3.31 ± 1.89^cd^	3.81 ± 0.29^abc^	2.90 ± 0.52^cd^	6.36 ± 0.15^abcd^
W_1_	N_1_	D_2_	0.95 ± 0.43	1.15 ± 0.20	1.00 ± 0.29	8.13 ± 0.97^a^	8.73 ± 0.23^abc^	5.94 ± 1.72^abc^	9.08 ± 0.04^a^	3.30 ± 1.14^abc^	4.06 ± 1.69^abcd^	6.86 ± 1.33^abc^
		D_3_	1.50 ± 0.81	1.33 ± 0.68	0.57 ± 0.09	1.50 ± 0.70^b^	3.77 ± 0.86^def^	6.39 ± 0.76^abc^	5.46 ± 0.05^bc^	4.65 ± 0.46^abc^	1.27 ± 0.14^d^	3.49 ± 0.29^def^
		D_1_	3.35 ± 0.26	2.10 ± 0.23	1.15 ± 0.26	1.35 ± 0.49^b^	6.05 ± 0.09^abcde^	7.84 ± 0.19^a^	3.93 ± 0.81^bcd^	4.42 ± 0.17^abc^	2.83 ± 1.48^cd^	1.10 ± 0.03^f^
	N_2_	D_2_	3.10 ± 1.04	2.40 ± 0.17	1.10 ± 0.12	2.70 ± 0.58^b^	9.10 ± 0.46^ab^	4.95 ± 1.42^abc^	5.10 ± 0.18^bcd^	5.25 ± 0.44^abc^	2.79 ± 0.07^cd^	4.03 ± 0.03^cdef^
		D_3_	2.65 ± 1.18	1.65 ± 0.43	2.55 ± 1.30	0.50 ± 0.12^b^	3.80 ± 0.58^def^	4.29 ± 0.20^abc^	2.62 ± 0.45^cd^	5.19 ± 1.09^abc^	8.78 ± 0.58^a^	3.51 ± 0.13^def^
		D_1_	0.97 ± 0.15	1.07 ± 0.44	1.30 ± 0.64	0.97 ± 0.43^b^	2.87 ± 0.92^def^	6.28 ± 0.56^abc^	3.84 ± 0.85^bcd^	5.15 ± 1.72^abc^	6.88 ± 0.80^abc^	7.19 ± 0.70^ab^
	N_0_	D_2_	1.15 ± 0.38	0.90 ± 0.42	1.25 ± 0.38	0.65 ± 0.09^b^	3.03 ± 0.20^def^	6.70 ± 0.30^ab^	5.99 ± 0.09^abc^	3.88 ± 0.32^abc^	5.43 ± 1.07^abcd^	1.63 ± 0.29^ef^
		D_3_	1.60 ± 0.12	1.20 ± 0.23	2.17 ± 0.20	1.30 ± 0.46^b^	2.53 ± 0.46^ef^	6.13 ± 0.46^abc^	3.75 ± 0.34^cd^	5.04 ± 0.44^abc^	6.43 ± 1.42^abc^	5.94 ± 0.25^abcd^
		D_1_	2.90 ± 0.92	2.15 ± 1.13	3.35 ± 0.32	1.65 ± 0.49^b^	4.53 ± 0.81^cdef^	1.87 ± 0.31^c^	6.02 ± 0.89^abc^	5.58 ± 1.37^abc^	2.42 ± 0.59^cd^	7.62 ± 0.53^a^
W_2_	N_1_	D_2_	3.25 ± 0.03	2.30 ± 0.64	2.25 ± 0.66	0.70 ± 0.12^b^	4.05 ± 0.26^def^	2.55 ± 0.61^bc^	4.03 ± 0.74^bcd^	2.03 ± 0.01^bc^	7.74 ± 0.81^ab^	4.36 ± 0.53^bcde^
		D_3_	1.65 ± 0.14	2.65 ± 0.03	0.95 ± 0.09	1.37 ± 0.15^b^	1.97 ± 0.59^ef^	2.24 ± 0.21^bc^	1.80 ± 0.12^d^	6.40 ± 1.57^ab^	3.96 ± 1.63^bcd^	7.06 ± 0.50^abc^
		D_1_	4.05 ± 0.14	0.90 ± 0.17	1.05 ± 0.20	1.40 ± 0.56^b^	9.30 ± 0.50^ab^	5.95 ± 0.71^abc^	4.33 ± 0.78^bcd^	1.19 ± 0.56^c^	1.38 ± 0.03^d^	6.39 ± 0.35^abcd^
	N_2_	D_2_	3.85 ± 0.55	3.35 ± 0.09	3.63 ± 0.12	2.20 ± 0.06^b^	5.971.92^abcde^	5.57 ± 1.89^abc^	9.14 ± 0.14^a^	6.18 ± 0.96^ab^	6.44 ± 0.47^abc^	4.84 ± 0.61^abcd^
		D_3_	3.63 ± 1.17	2.00 ± 0.12	3.30 ± 0.06	2.05 ± 0.72^b^	7.05 ± 1.24^abcd^	7.63 ± 0.74^a^	7.35 ± 0.04^ab^	7.44 ± 0.49^a^	3.73 ± 0.16^bcd^	5.25 ± 0.47^abcd^
*p*-value			0.230	0.483	0.193	0.012	0.020	0.019	<0.001	0.011	<0.001	0.004
Lsd			1.71	1.26	1.20	1.89	2.38	2.50	1.93	2.53	2.61	1.68
**Water level **	**N rate**	**Plant density**	**150 days after planting and soil depth (2018)**					**150 days after planting and soil depth (2019)**				
			**0–30 cm**	**30–60 cm**	**60–90 cm**	**90–120 cm**	**120–150 cm**	**0–30 cm**	**30–60 cm**	**60–90 cm**	**90–120 cm**	**120–150 cm**
		D_1_	1.25 ± 0.61^d^	0.50 ± 0.06	1.55 ± 0.38^cdef^	1.85 ± 0.14	0.80 ± 0.21	7.29 ± 0.81	4.33 ± 0.25^bcde^	3.52 ± 0.18	3.24 ± 0.54^cde^	7.64 ± 0.20
	N_0_	D_2_	3.30 ± 0.64^abcd^	1.40 ± 0.12	1.50 ± 0.23^cdef^	1.80 ± 0.17	0.25 ± 0.10	7.77 ± 0.13	6.26 ± 0.05^abcd^	2.90 ± 0.88	2.84 ± 0.108^de^	7.11 ± 1.15
		D_3_	3.95 ± 0.09^abcd^	2.30 ± 0.35	0.85 ± 0.26^ef^	1.60 ± 0.12	1.57 ± 0.29	7.27 ± 0.71	6.71 ± 0.87^abc^	3.22 ± 1.05	3.57 ± 0.23^bcde^	5.58 ± 0.89
		D_1_	4.30 ± 0.87^abc^	1.97 ± 0.41	0.45 ± 0.03^f^	1.65 ± 0.09	1.40 ± 0.25	6.25 ± 1.05	4.99 ± 1.89^abcde^	3.18 ± 0.29	3.02 ± 0.52^cde^	6.05 ± 0.15
W_1_	N_1_	D_2_	4.10 ± 0.23^abcd^	2.25 ± 0.38	2.50 ± 0.92^abcde^	1.35 ± 0.14	1.60 ± 0.66	6.82 ± 1.72	6.92 ± 0.04^abc^	2.22 ± 1.14	1.47 ± 1.69^e^	2.09 ± 1.33
		D_3_	4.10 ± 1.01^abcd^	2.57 ± 1.25	2.60 ± 0.21^abcd^	1.33 ± 0.37	1.23 ± 0.70	4.52 ± 0.76	4.21 ± 0.05^bcde^	4.91 ± 0.46	2.67 ± 0.14^de^	5.29 ± 0.29
		D_1_	5.35 ± 0.26^ab^	2.50 ± 0.12	0.90 ± 0.29^ef^	1.50 ± 0.06	1.53 ± 0.25	7.18 ± 0.19	3.83 ± 0.81^bcde^	7.61 ± 0.17	5.80 ± 1.48^abc^	3.91 ± 0.03
	N_2_	D_2_	4.10 ± 0.81^abcd^	2.85 ± 0.84	1.75 ± 0.26^bcdef^	2.10 ± 0.12	1.32 ± 0.29	7.16 ± 1.42	5.38 ± 0.18^abcde^	7.18 ± 0.44	4.43 ± 0.07^bcde^	4.57 ± 0.03
		D_3_	5.40 ± 017^ab^	1.35 ± 0.32	1.05 ± 0.26def	1.75 ± 0.03	0.87 ± 0.37	5.03 ± 0.20	2.36 ± 0.45^de^	7.78 ± 1.09	2.47 ± 0.58^de^	6.76 ± 0.13
		D_1_	2.15 ± 0.43^cd^	2.15 ± 0.14	2.30 ± 0.06^abcde^	1.60 ± 0.06	1.53 ± 0.52	7.71 ± 0.56	7.28 ± 0.85^abc^	5.68 ± 1.72	4.89 ± 0.80^bcd^	6.05 ± 0.70
	N_0_	D_2_	2.10 ± 0.35^cd^	3.55 ± 0.78	2.15 ± 0.20^abcde^	1.65 ± 0.03	1.20 ± 0.56	8.02 ± 0.30	7.93 ± 0.09^ab^	6.26 ± 0.32	3.46 ± 1.07^bcde^	5.68 ± 0.29
		D_3_	2.55 ± 0.72^bcd^	1.30 ± 0.06	2.15 ± 0.03^abcde^	1.35 ± 0.66	0.77 ± 0.18	5.52 ± 0.46	9.14 ± 0.34^a^	3.54 ± 0.44	3.02 ± 1.42^cde^	2.31 ± 0.25
		D_1_	2.85 ± 0.89^abcd^	2.55 ± 0.78	1.80 ± 0.35^bcdef^	0.40 ± 0.17	2.90 ± 0.45	8.77 ± 0.31	8.73 ± 0.89^a^	5.96 ± 1.37	4.16 ± 0.59^bcde^	3.09 ± 0.53
W_2_	N_1_	D_2_	5.75 ± 0.09^a^	3.10 ± 1.03	1.00 ± 0.23^def^	0.90 ± 0.29	2.12 ± 0.77	5.60 ± 0.61	3.10 ± 0.74^cde^	6.12 ± 0.01	8.33 ± 0.81^a^	5.06 ± 0.53
		D_3_	3.70 ± 0.64^abcd^	2.35 ± 0.43	2.75 ± 0.26^abc^	0.35 ± 0.09	4.67 ± 1.09	8.76 ± 0.21	5.11 ± 0.12^abcde^	6.15 ± 1.57	4.34 ± 1.63^bcde^	3.32 ± 0.50
		D_1_	4.25 ± 0.26^abc^	3.20 ± 0.52	2.85 ± 0.32^abc^	1.70 ± 0.06	4.65 ± 1.40	5.96 ± 0.71	7.41 ± 0.78^ab^	6.34 ± 0.56	6.31 ± 0.03^ab^	2.69 ± 0.35
	N_2_	D_2_	1.95 ± 0.38^cd^	4.00 ± 0.62	3.70 ± 0.12^a^	1.65 ± 0.09	3.00 ± 1.23	6.69 ± 1.89	1.56 ± 0.14^e^	4.57 ± 0.96	2.63 ± 0.47^de^	1.68 ± 0.61
		D_3_	3.10 ± 0.00^abcd^	3.95 ± 0.32	3.25 ± 0.26^ab^	2.17 ± 0.15	5.09 ± 0.89	6.01 ± 0.74	3.85 ± 0.04^bcde^	4.91 ± 0.49	3.91 ± 0.16^bcde^	1.92 ± 0.47
*p*-value			0.027	0.054	0.022	0.135	0.125	0.089	0.046	0.705	<0.001	0.061
Lsd			1.60	1.67	0.92	0.62	1.95	2.81	2.28	2.34	1.60	2.59

**Note:**

Irrigation (water level) comprised of W_1_ = 5,250 m^3^/hm^2^ and W_2_ = 4,740 m^3^/hm^2^, nitrogen fertilizer consisted of N_0_ = 0 kg N ha^−1^, N_1_ = 270 kg N ha^−1^, and N_2_ = 360 kg N ha^−1^ and maize plant density included D_1_ = 75,000 plants/ha^−1^, D_2_ = 97,500 plants/ha^−1^, and D_3_ = 120,000 plants/ha^−1^. Values (mean ± standard error of mean) with different superscripts in the same column are significantly different at *p* < 0.05.

It was also observed that accumulation of nitrate generally decreased as the soil depth increased to a depth of 60–90 cm where it increased thereafter for planting duration of 30 days to 120 days (Figs. 2A & 2D). Conversely, nitrate accumulation in the soil generally declined as soil depth advanced at 150 days after planting (Fig. 2E). Nonetheless, more accumulation was shown to have occurred at the extreme soil depths at certain durations after planting. For example, higher nitrate accumulation occurred at 0–30 cm for 30 days, 90 days, and 150 days after planting while higher values were recorded at a soil depth of 120–150 cm for 60 days and 120 days after planting in 2018. No such trends were however recorded for soil depth for the various factor interactions. It was further observed that leaching in 2019 was more pronounced than in 2018.

### Ammonia emission on maize farmland as affected by nitrogen fertilization, plant density and irrigation, and their interactions

**Figure 3:**
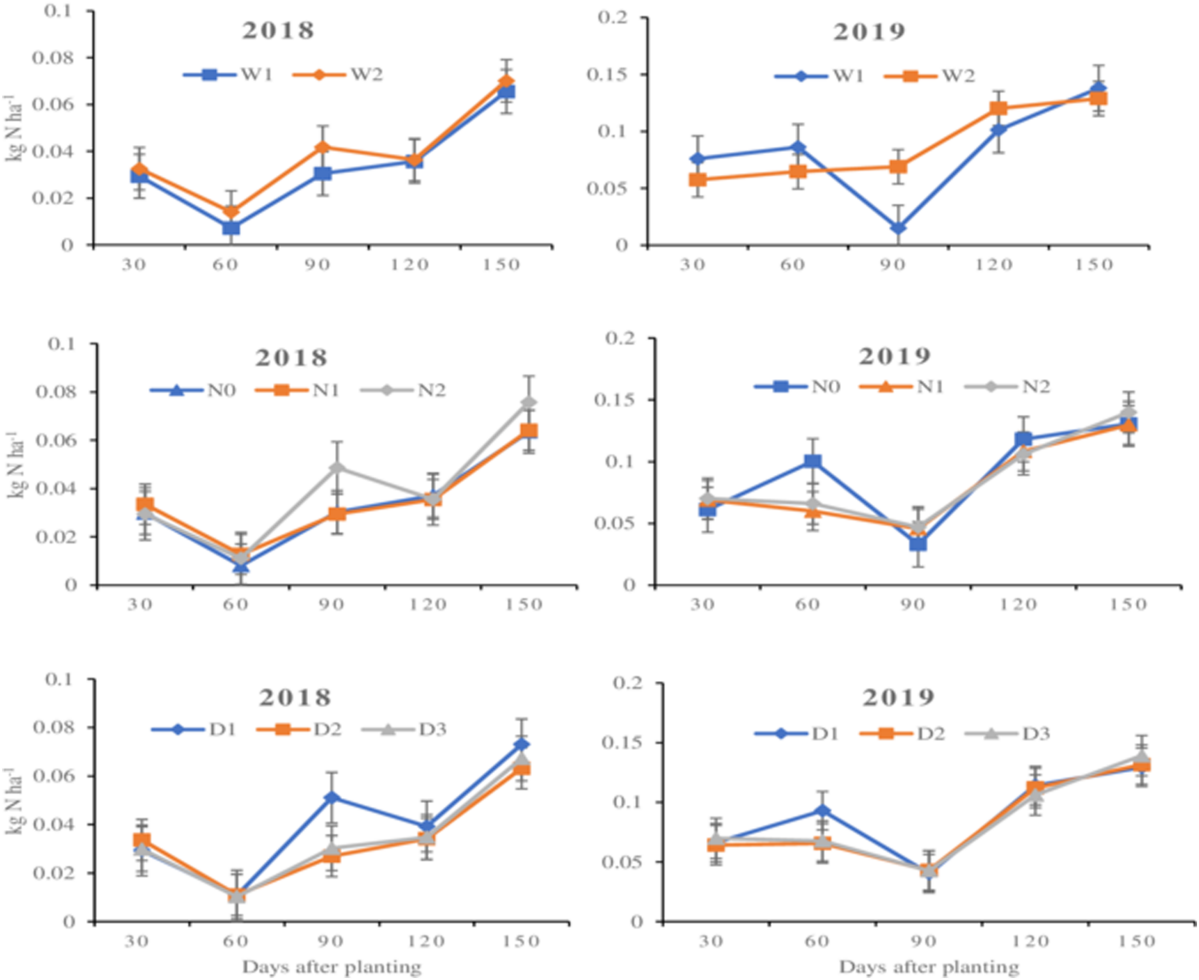
Influence of individual treatment levels on NH_3_–N emission across the experimental years. Influence of individual treatment levels on NH_3_–N emission across the experimental years. (A, B) Represent irrigation (water level) in 2018 and 2019, respectively, where W_1_ = 5,250 m^3^/hm^2^ and W_2_ = 4,740 m^3^/hm^2^. (C, D) Represent N fertilizer levels in 2018 and 2019, respectively, where N_0_ = 0 kg N ha^−1^, N_1_ = 270 kg N ha^−1^, and N_2_ = 360 kg N ha^−1^. (E, F) Represent maize plant density in 2018 and 2019, respectively, D_1_ = 75,000 plants/ha^−1^, D_2_ = 97,500 plants/ha^−1^, and D_3_ = 120,000 plants/ha^−1^. The error bars represent standard error of means from the triplicate readings.

The influence of N rate, plant density and irrigation level on ammonia emission is presented in Fig. 3 and Tables 6 and 7. It was found that ammonia emission was significantly (*p* < 0.05) affected by irrigation level at 60 days after planting in 2018 and at 30 days, 90 days, and 120 days after planting in 2019 with a general decrease as irrigation level increased (Fig. 3). Nitrogen rate did not have any significant influence on ammonia emission in 2018 but markedly affected emission at (*p* < 0.05) at 90 days and 120 days after planting in 2019. The quantity of ammonia emitted averagely increased as more fertilizer was applied especially at 90 days and 150 days in 2018 and at 30 days, 90 days and 150 days after planting in 2019 (Fig. 3). Plant density alone did not have any significant effect (*p* > 0.05) on the amount of ammonia emitted irrespective of the duration after planting and the year of cultivation even though D_1_ (75,000 plants/ha^−1^) relatively recorded the highest emission of ammonia as compared to the higher plant densities (Fig. 3).

**Table 6:** Combined effect of N fertilizer rates and irrigation (water level) on NH_3_–N emissions across the 2 years on maize farmland. Nitrogen fertilizer rates consisted of N_0_ = 0 kg N ha^−1^, N_1_ = 270 kg N ha^−1^, and N_2_ = 360 kg N ha^−1^. Irrigation (water levels) comprised W_1_ = 5,250 m^3^/hm^2^ and W_2_ = 4,740 m^3^/hm^2^. Means separation was done with least significant difference at *p*-value < 0.05. Means ± SEMs with a common superscript indicates no significant difference at *p* < 0.05 and those with otherwise are significantly different at *p* < 0.05.

Water level	N fertilizer level	Days after planting (2018)					Days after planting (2019)				
		30	60	90	120	150	30	60	90	120	150
W_1_	N_0_	0.032 ± 0.01	0.002 ± 0.00	0.034 ± 0.00	0.043 ± 0.01^a^	0.063 ± 0.01	0.069 ± 0.00	0.132 ± 0.06	0.017 ± 0.00^a^	0.113 ± 0.01	0.133 ± 0.00
	N_1_	0.030 ± 0.00	0.011 ± 0.00	0.032 ± 0.00	0.040 ± 0.00^a^	0.060 ± 0.00	0.077 ± 0.00	0.062 ± 0.00	0.014 ± 0.00^a^	0.098 ± 0.00	0.132 ± 0.00
	N_2_	0.026 ± 0.00	0.008 ± 0.00	0.026 ± 0.00	0.024 ± 0.00^b^	0.073 ± 0.00	0.082 ± 0.00	0.064 ± 0.00	0.014 ± 0.00^a^	0.092 ± 0.00	0.149 ± 0.02
W_2_	N_0_	0.028 ± 0.00	0.014 ± 0.00	0.027 ± 0.00	0.031 ± 0.00^ab^	0.064 ± 0.01	0.053 ± 0.01	0.069 ± 0.00	0.049 ± 0.01^b^	0.123 ± 0.01	0.128 ± 0.00
	N_1_	0.037 ± 0.01	0.014 ± 0.00	0.027 ± 0.00	0.031 ± 0.00^ab^	0.068 ± 0.00	0.061 ± 0.01	0.058 ± 0.00	0.078 ± 0.00^c^	0.118 ± 0.00	0.127 ± 0.00
	N_2_	0.033 ± 0.01	0.014 ± 0.00	0.072 ± 0.04	0.047 ± 0.00^a^	0.078 ± 0.01	0.059 ± 0.00	0.067 ± 0.01	0.080 ± 0.00^c^	0.120 ± 0.01	0.131 ± 0.00
*p*-value		0.484	0.058	0.286	<0.001	0.814	0.704	0.340	0.001	0.193	0.567
Lsd		0.014	0.005	0.054	0.013	0.017	0.013	0.070	0.014	0.014	0.021

**Note:**

Nitrogen fertilizer rates consisted of N_0_ = 0 kg N ha^−1^, N_1_ = 270 kg N ha^−1^, and N_2_ = 360 kg N ha^−1^. Irrigation (water levels) comprised W_1_ = 5,250 m^3^/hm^2^ and W_2_ = 4,740 m^3^/hm^2^. Values (mean ± standard error of mean) with different superscripts in the same column are significantly different at *p* < 0.05.

**Table 7:** Combined effect of plant density and irrigation (water level) on NH_3_–N emission across the 2 years in maize farmland. Plant density included D_1_ = 75,000 plants/ha^−1^, D_2_ = 97,500 plants/ha^−1^, and D_3_ = 120,000 plants/ha^−1^. Irrigation (water level) comprised W_1_ = 5,250 m^3^/hm^2^ and W_2_ = 4,740 m^3^/hm^2^. Means separation was done with least significant difference at *p*-value < 0.05. Means ± SEMs with a common superscript indicates no significant difference at *p* < 0.05 and those with otherwise are significantly different at *p* < 0.05.

Water level	N rate	Plant density	Days after planting (2018)					Days after planting (2019)				
			30 days	60 days	90 days	120 days	150 days	30 days	60 days	90 days	120 days	150 days
		D_1_	0.031 ± 0.01	0.003 ± 0.00	0.033 ± 0.00	0.059 ± 0.02	0.090 ± 0.03^a^	0.066 ± 0.00	0.248 ± 0.17	0.018 ± 0.00^c^	0.115 ± 0.02	0.135 ± 0.00
	N_0_	D_2_	0.031 ± 0.01	0.002 ± 0.00	0.026 ± 0.00	0.029 ± 0.01	0.068 ± 0.01^ab^	0.073 ± 0.00	0.066 ± 0.00	0.022 ± 0.01^bc^	0.118 ± 0.01	0.133 ± 0.00
		D_3_	0.033 ± 0.01	0.001 ± 0.00	0.042 ± 0.01	0.042 ± 0.01	0.033 ± 0.01^b^	0.069 ± 0.01	0.083 ± 0.01	0.013 ± 0.01^c^	0.107 ± 0.00	0.130 ± 0.00
		D_1_	0.025 ± 0.01	0.010 ± 0.00	0.037 ± 0.01	0.041 ± 0.00	0.053 ± 0.01^ab^	0.080 ± 0.01	0.052 ± 0.01	0.013 ± 0.00^c^	0.096 ± 0.01	0.133 ± 0.00
W_1_	N_1_	D_2_	0.033 ± 0.01	0.017 ± 0.00	0.034 ± 0.01	0.036 ± 0.00	0.058 ± 0.00^ab^	0.075 ± 0.00	0.066 ± 0.00	0.017 ± 0.00^c^	0.104 ± 0.00	0.132 ± 0.00
		D_3_	0.032 ± 0.01	0.007 ± 0.00	0.025 ± 0.00	0.042 ± 0.00	0.069 ± 0.01^ab^	0.075 ± 0.01	0.069 ± 0.00	0.012 ± 0.00^c^	0.094 ± 0.01	0.132 ± 0.00
		D_1_	0.028 ± 0.00	0.014 ± 0.01	0.023 ± 0.00	0.025 ± 0.00	0.077 ± 0.00^ab^	0.075 ± 0.01	0.068 ± 0.01	0.010 ± 0.00^c^	0.098 ± 0.00	0.130 ± 0.00
	N_2_	D_2_	0.024 ± 0.01	0.005 ± 0.00	0.023 ± 0.00	0.027 ± 0.00	0.066 ± 0.01^ab^	0.083 ± 0.01	0.066 ± 0.01	0.013 ± 0.00^c^	0.090 ± 0.01	0.133 ± 0.00
		D_3_	0.026 ± 0.01	0.005 ± 0.00	0.031 ± 0.00	0.019 ± 0.01	0.077 ± 0.00^ab^	0.087 ± 0.00	0.059 ± 0.01	0.017 ± 0.01^c^	0.089 ± 0.00	0.184 ± 0.01
		D_1_	0.016 ± 0.00	0.012 ± 0.00	0.029 ± 0.01	0.031 ± 0.01	0.065 ± 0.01^ab^	0.047 ± 0.01	0.068 ± 0.00	0.022 ± 0.01^bc^	0.123 ± 0.01	0.125 ± 0.00
	N_0_	D_2_	0.043 ± 0.01	0.013 ± 0.00	0.018 ± 0.01	0.027 ± 0.01	0.044 ± 0.02^ab^	0.047 ± 0.01	0.074 ± 0.01	0.062 ± 0.02^ab^	0.127 ± 0.01	0.130 ± 0.00
		D_3_	0.025 ± 0.01	0.017 ± 0.01	0.033 ± 0.00	0.035 ± 0.01	0.082 ± 0.01^ab^	0.064 ± 0.02	0.064 ± 0.01	0.063 ± 0.03^ab^	0.118 ± 0.00	0.131 ± 0.00
		D_1_	0.033 ± 0.01	0.012 ± 0.01	0.032 ± 0.01	0.037 ± 0.00	0.081 ± 0.01^ab^	0.071 ± 0.01	0.060 ± 0.00	0.088 ± 0.00^a^	0.119 ± 0.01	0.123 ± 0.00
W_2_	N_1_	D_2_	0.031 ± 0.01	0.016 ± 0.00	0.025 ± 0.00	0.036 ± 0.01	0.060 ± 0.01^ab^	0.047 ± 0.00	0.054 ± 0.00	0.067 ± 0.00^a^	0.123 ± 0.01	0.130 ± 0.00
		D_3_	0.047 ± 0.01	0.014 ± 0.00	0.023 ± 0.01	0.020 ± 0.01	0.064 ± 0.00^ab^	0.065 ± 0.00	0.060 ± 0.00	0.078 ± 0.00^a^	0.114 ± 0.01	0.128 ± 0.00
		D_1_	0.042 ± 0.01	0.012 ± 0.01	0.152 ± 0.13	0.043 ± 0.00	0.072 ± 0.01^ab^	0.058 ± 0.00	0.062 ± 0.00	0.091 ± 0.00^a^	0.133 ± 0.02	0.131 ± 0.00
	N_2_	D_2_	0.041 ± 0.01	0.013 ± 0.01	0.036 ± 0.00	0.049 ± 0.01	0.083 ± 0.01^ab^	0.060 ± 0.00	0.069 ± 0.01	0.077 ± 0.00^a^	0.110 ± 0.01	0.131 ± 0.00
		D_3_	0.016 ± 0.01	0.018 ± 0.00	0.027 ± 0.01	0.050 ± 0.00	0.080 ± 0.01^ab^	0.058 ± 0.00	0.071 ± 0.00	0.073 ± 0.01^a^	0.116 ± 0.01	0.130 ± 0.00
*p*-value			0.267	0.600	0.531	0.359	0.005	0.683	0.412	0.021	0.977	0.312
Lsd			0.025	0.009	0.093	0.022	0.030	0.022	0.122	0.023	0.024	0.036

**Note:**

Nitrogen fertilizer rates consisted of N_0_ = 0 kg N ha^−1^, N_1_ = 270 kg N ha^−1^, and N_2_ = 360 kg N ha^−1^. Irrigation (water levels) comprised W_1_ = 5,250 m^3^/hm^2^ and W_2_ = 4,740 m^3^/hm^2^. Values (mean ± Standard error of mean) with different superscripts in the same column are significantly different at *p* < 0.05.

Interactively, the combination of N rate and irrigation level affected emission (*p* < 0.05) at 120 days and 90 days after planting in 2018 and 2019, respectively (Table 6). Regardless, there was an overall decrease in emission as more N rate was applied at the higher irrigation level (W_1_) while an increase was rather observed as N rate increased and irrigation level lowered. The combination of irrigation level and plant density as well as that of N rate and plant density however did not have any substantial influence (*p* > 0.05) on ammonia emission irrespective of the days after planting. In terms of three-factor influence, marked differences only existed at 150 days after planting in 2018 and at 90 days after planting in 2019 (Table 7). At 150 days after planting in 2018, the lowest (0.033 kg N ha^−1^) emission occurred when zero N rate was applied to the highest plant density irrigated at 5,250 m^3^/hm^2^ (W_1_ × N_0_ × D_3_) while the highest (0.090 kg N ha^−1^) was recorded by W_1_ × N_0_ × D_1_. For the 90 days after planting in 2019, the lowest (0.010 kg N ha^−1^) emission was recorded at W_1_ × N_1_ × D_1_ and the highest (0.91 kg N ha^−1^) was recorded at W_2_ × N_2_ × D_1_. Nonetheless, the overall lowest (0.001 kg N ha^−1^) emission however occurred on W_1_ × N_0_ × D_3_ at 60 days after planting in 2018 while the highest (0.248 kg N ha^−1^) was recorded on W_1_ × N_0_ × D_1_ at 60 days after planting in 2019. It could also be observed that emission relatively increased with an increase in duration after planting except at 60 days in 2018 and 90 days in 2019. It could further be observed that ammonia emission in 2019 was relatively higher than that recorded in 2018.

## Discussion

Anthropogenic activities, especially excess synthetic N fertilizer application in agriculture production systems contribute significantly to nitric oxide (NO and NO_2_), ammonia (NH_3_–N), and nitrous oxide (N_2_O) release which are major contributors to global warming (Akbari et al., 2020). The rate of N application influences its availability in agricultural soils. Excessive use of N has led to numerous environmental problems, such as acidification of soils, gaseous emissions, and eutrophication of water resources (Khakbazan et al., 2014; Kong et al., 2017).

The varying amounts of ammonia, nitrate and total nitrogen in the soil recorded between 2018 and 2019 before the commencement of the experiment is an indication of how the year of cultivation can influence these nutrients as a result of variation in soil properties and climatic conditions Table 1 and Fig. 1. The initial amounts of ammonia and nitrate in the soil before the experiment may largely contribute to the relatively higher amount of nitrate accumulation in the soil and the higher ammonia emission recorded in 2019 as compared to 2018 during the experiment. The rate of nitrogen applied in the previous year, the type of crop grown, and the precipitation amount can influence the availability of soil nitrogen (Chatterjee, 2020). More N fertilization, less uptake by crops and accumlulated N over the years facilitates higher leaching and emission of N, as observed in this experiment.

Increased irrigation level, has the tendency to accumulate NO_3_-N in deeper soil layers, as also observed by Xiang et al., 2019. However, the overall increased accumulation and leaching when a lower (W_2_) irrigation level was applied and may not entirely be out of order as similar findings were also reported elsewhere by Rong & Xuefeng (2011). Poor soil structure and physical properties (Rengasamy, 2010) could explain the relatively lower leached of N nitrate recorded by W_1_. The increase in accumulation of nitrate in the soil with increasing N fertilizer rate corroborates with findings of Verma & Sagar (2020), and Rong & Xuefeng (2011). This is because increasing the amount of N fertilizer applied may exceed the amount required by the crops, hence leading to increased quantities being accumulated and leached into deeper soil layers (Xu et al., 2020).

Similarly, the relatively higher accumulation of nitrate in the soil at the lower plant densities goes to buttress the point that when N fertilization exceeds demand by plants, accumulation of the fertilizer increases in soil. This is because the lesser the plant density, the lower the fertilization demand and *vice versa*. High accumulation of the nitrate in the soil measured at 30 days after planting could be attributed to low uptake by the young plants due to less-developed root systems (Qingfeng et al., 2016). In terms of soil depth, the accumulation of nitrate in 0–30 cm shows nutrient’s availability within the root zone of the maize plants. The accumulation of the nitrate in deeper soil depths especially in 120–150 cm conversely shows the nutrient is beyond the reach of many shallow-rooted crops like maize which can negatively affects their growth and yield.

Ammonium fertilizers easily emit nitrogen gases into the atmosphere as a result of the conversion of the ammonium to ammonia (NH_3_–N) gas (Wang, Köbke & Dittert, 2020). The relatively higher emission occurring at the lower irrigation level could be related to the relatively lower amount of water available to reduce ammonia emission. This finding agrees with Han, Zhou & Wang (2014), Han et al. (2016). However, ammonia emission can be partly influenced by soil properties such as moisture content, temperature and soil texture (Dutta et al., 2018), Fig. 1 and Table 1. Warmer temperatures, coarse soil texture, precipitation, and high moisture content facilitates emission of ammonia and *vice versa*. Therefore, the variations in precipitation and air temperature as recorded in this study (Fig. 1) explains the variations in emission of ammonia recorded in the study.

Increased quantities of N fertilization especially at the surface of the soil increases ammonia emission which best explain the relatively increased emission as the N rate increases. Furthermore, the significant effect of irrigation level and N rate at specific durations after planting is an indication that ammonia emission as triggered by these factors is time-dependent. The insignificant effect by plant density on ammonia emission shows that plant density does not affect ammonia emission. Also, the non-linear trends in ammonia emission as influenced by the factor interactions is an indication that combining increased or decreased amounts of the treatments may not correspondingly affect ammonia emission. Nevertheless, increasing duration after planting will correspondingly lead to more emission of ammonia as more emission will likely occur. Both nitrate leaching and ammonia emission result in loss of nitrogen, making it unavailable for plant use, hence negatively affecting agriculture productivity (Wang et al., 2017).

## Conclusions

The results showed that irrigation level, nitrogen fertilizer rate and plant density significantly affected leaching and accumulation of nitrate individually and interactively irrespective of the soil depth, days after planting and year of cultivation. The results also showed that factor combinations did not result in linear trends of leaching and accumulation of nitrate and emission. Higher accumulation occurred at deeper soil depths except at 150 days after planting where nitrate accumulation in the soil declined as soil depth increased. Irrigation level and nitrogen rate affected emission of ammonia at 30, 60, 90, and 120 days after planting in 2018 and 2019. Ammonia mission decreased with an increase in irrigation level but increased with an increase in N rate.

A significant effect of the combination of all three factors was only recorded at 150 days after planting for both years. However, 0 kg N ha^−1^ N rate at 120,000 plants/ha^−1^ irrigated with 5,250 m^3^/hm^2^ caused the lowest emission in 2018 while 0 kg N ha^−1^ and plant density at 75,000 plants/ha^−1^ irrigated with 5,250 m^3^/hm^2^ caused the highest emission in 2019 both at 60 days after planting. These findings, therefore suggests, it is possible to optimize nitrogen rate, plant density and irrigation level to reduce nitrogen losses through nitrate leaching and ammonia emission, as was achieved in this study, for agricultural and environmental sustainability.

## Supplemental Information

10.7717/peerj.12762/supp-1 Supplemental Information 1 NH3 2018 raw data during sampling.

10.7717/peerj.12762/supp-2 Supplemental Information 2 NH3 2018 emission at sampling. NH3 sampling raw data of 2019

10.7717/peerj.12762/supp-3 Supplemental Information 3 NO3 levels raw data of 2018. NO3 levels and accumulation at sampling

10.7717/peerj.12762/supp-4 Supplemental Information 4 NO3 raw data 2019. NO3 levels at sampling in 2019

10.7717/peerj.12762/supp-5 Supplemental Information 5 Air temperature and precipiation raw data at station. Raw data of air temperature and precipiation of 2018 and 2019

## Additional Information and Declarations

### Data Availability

The following information was supplied regarding data availability: The raw data is available in the Supplemental Files.
